# CTCF induces histone variant incorporation, erases the H3K27me3 histone mark and opens chromatin

**DOI:** 10.1093/nar/gku937

**Published:** 2014-10-07

**Authors:** Oliver Weth, Christine Paprotka, Katharina Günther, Astrid Schulte, Manuel Baierl, Joerg Leers, Niels Galjart, Rainer Renkawitz

**Affiliations:** 1 Institute for Genetics, Justus-Liebig-University, 35392 Giessen, Germany; 2 Department of Cell Biology and Genetics, Erasmus MC, 3000 CA Rotterdam, The Netherlands

## Abstract

Insulators functionally separate active chromatin domains from inactive ones. The insulator factor, CTCF, has been found to bind to boundaries and to mediate insulator function. CTCF binding sites are depleted for the histone modification H3K27me3 and are enriched for the histone variant H3.3. In order to determine whether demethylation of H3K27me3 and H3.3 incorporation are a requirement for CTCF binding at domain boundaries or whether CTCF causes these changes, we made use of the LacI DNA binding domain to control CTCF binding by the Lac inducer IPTG. Here we show that, in contrast to the related factor CTCFL, the N-terminus plus zinc finger domain of CTCF is sufficient to open compact chromatin rapidly. This is preceded by incorporation of the histone variant H3.3, which thereby removes the H3K27me3 mark. This demonstrates the causal role for CTCF in generating the chromatin features found at insulators. Thereby, spreading of a histone modification from one domain through the insulator into the neighbouring domain is inhibited.

## INTRODUCTION

On the 2D level the eukaryotic genome is structured into domains, which may serve several functions. One of these functions is to group euchromatic and heterochromatic genomic regions into separate domains. In fact, heterochromatic domains are significantly expanded in the human genome of fibroblasts as compared to embryonic stem cells (1). As a marker for a repressed domain the triple methylation of lysine27 of histone H3 (H3K27me3) is often found, which is a hallmark of Polycomb-repressed chromatin (2,3). The absence of such a mark and the presence of methylated H3K4 or acetylated H3K9 are indicative of an active domain. Thus, one definition of a domain border is the switch from repressive marks to active marks on the chromatin landscape. Insulators, often characterized by active chromatin marks (4,5), functionally separate domains from each other and ensure the proper maintenance of activity status. The insulator factor, CTCF, has been found in a subset of domain boundaries (6–12). Another definition of domain boundaries is given by the ability to generate 3D chromatin loops. The characterization of the CTCF bound chromatin interactome has identified loop contacts associated with CTCF binding (11). Again, these interacting CTCF boundaries showed a unique enrichment for H3K27me3 within the loops. Finally, the unbiased search for interacting domain boundaries using the Hi-C technique (13) within the mouse, human and *Drosophila* genomes has revealed local chromatin domains defined by a higher frequency of chromatin contacts within the domain as compared to outside regions (14,15). Again, a subset of these boundaries is bound by CTCF with a clear segregation of repressive and active chromatin marks at these regions.

These results indicate that among the many cases of domain boundaries a subpopulation is marked by CTCF binding (11,12,16). For HeLa cells, 793 domain boundaries show CTCF binding (10). Of these, 12 sites are framing H3K27me3 domains on both sides. Very likely, additional factors besides CTCF are involved in boundary function, as exemplified by the composite boundary of the chicken β-globin locus (17,18). In case of CTCF sites, this factor might prevent the spread of the repressive mark into a flanking domain. On a global scale, depletion of CTCF results in a small change of H3K27me3 spreading (19), as there are many more CTCF sites with other functions besides a boundary function. In contrast, when analysing individual genes, knockdown of CTCF (20,21), or of a CTCF deficiency mutant (22), resulted in an extended H3K27me3 mark into the flanking region. Similarly, homeotic gene clusters in mouse and Drosophila are inactivated within H3K27me3 domains, which are framed by CTCF (23,24). In case of the HoxA cluster, depletion of CTCF causes an increase in H3K27me3 at the CTCF site (24). Opposing effects have been observed as well, i.e. depletion of CTCF also caused the spreading from an active domain into the flanking repressed region as judged from a reduction of the H3K27me3 levels (20,25).

The question remained, how does CTCF mechanistically prevent the spreading of chromatin marks beyond a boundary? The boundary regions themselves are depleted for the repressive mark H3K27me3 and are enriched for active marks (14). Is H3K27me3 depletion, i.e. demethylation of H3K27, a requirement for CTCF binding and/or function, or is CTCF causing such a demethylation? Furthermore, the enrichment for the variant histone H3.3 at CTCF sites (26) has been discussed as a requirement for CTCF binding (27). Again, a causal relationship has not been demonstrated. To address these questions we uncoupled DNA binding from CTCF function by fusing CTCF to the DNA-binding domain of the Lac-repressor. Here, we find that targeting of CTCF to a heterochromatic LacO repeat cluster causes a rapid chromatin demethylation of H3K27me3 followed by a dramatic chromatin de-condensation. Histone demethylation is accompanied by transient incorporation of the histone variant H3.3, suggesting that histone exchange is the mechanism for histone demethylation, and that this may be the mechanism to prevent spreading of histone marks from one domain through the insulator into the neighbouring domain.

## MATERIALS AND METHODS

### Cell culture and transfection

The clonal cell line U2OS with stably integrated LacO array (F42B8 and F6B8, Karsten Rippe, Heidelberg, Germany), HeLa as well as HeLa S3 Flag-HA-H3.3 / H3.1 cells were cultured at 37°C with 5% CO_2_ in Dulbecco's modified Eagle's medium supplemented with 10% (v/v) serum and 1% PenStrep.

For immunostaining U2OS cells were seeded onto glass coverslips at the bottom of 6-well plates and allowed to grow for 24 h before transfection. If required, 150 μM isopropyl β-D-1-thiogalactopyranoside (IPTG) was added before transfection and removed by washing twice with phosphate buffered saline (PBS) for the indicated time periods. Subsequently, cells were transfected with JetPEI (Polyplus transfection) essentially as described in the manual. In detail, 1 μg of LacI constructs and 2 μg pBSK were used with 6 μl of JetPEI per well. Fresh medium was added after 4 h and the cells were further incubated for 48 h followed by immunostaining.

### Immunocytochemistry

For immunofluorescence analysis cells were washed once with PBS and fixed with 4% paraformaldehyde for 10 min. After fixation cells were washed twice with PBS, permeabilized for 10 min in 0.15% Triton (PBS, 0.15%Triton X-100) and blocked for 1 h in 2% bovine serum albumin (BSA) (2% BSA, 0.05% Tween-20, PBS) followed by incubation for 1 h with primary antibodies diluted in blocking buffer (H3K27me3, 1:300, Millipore 07-449; H3K9ac, 1:200, Upstate 07-352; H4ac, 1:200, Upstate 06-866). Afterwards the coverslips were rinsed three times in PBS and incubated for 1 h with secondary antibody diluted in blocking buffer (Invitrogen A11011, 1:200). Cells were then washed three times with PBS and stained with 2.5 μg/ml Hoechst for 10 min followed by one PBS rinse. Coverslips were mounted in Fluromount and analysed with a microscope. Size and intensity of detected arrays were measured using the Volocity software.

### Chromatin immunoprecipitation (ChIP) and native ChIP, RNA isolation, luciferase assay, western blot, Formaldehyde-Assisted Isolation of Regulatory Elements (FAIRE), plasmids and primer

See Supplementary Materials and Methods.

## RESULTS

### Boundary model sites are devoid of H3K27me3 and require CTCF to function

**Figure 1.**
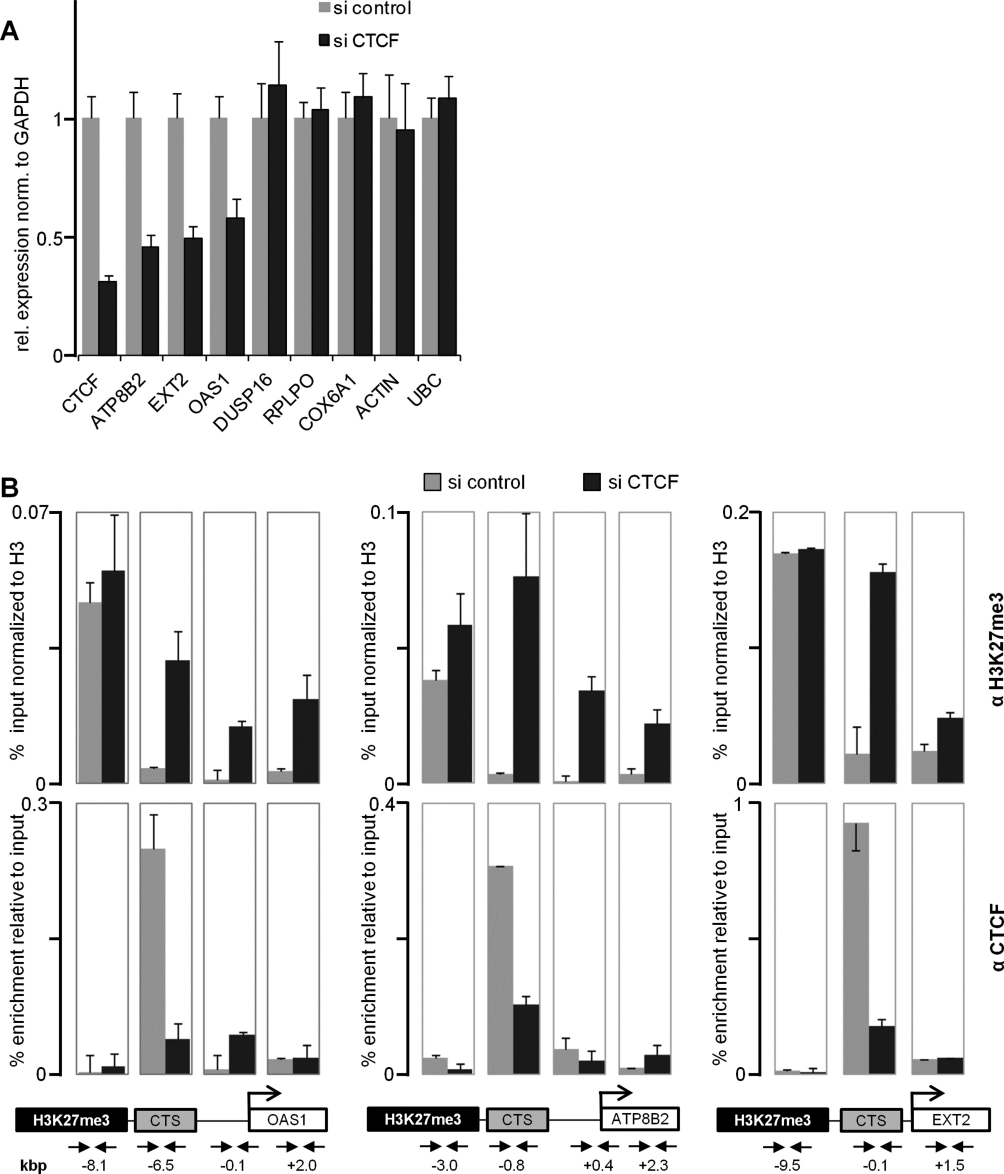
CTCF barrier sites show a spreading of H3K27me3 signal upon depletion of CTCF. HeLa cells were transfected with either CTCF or control siRNA. (A) After 6 days RNA was isolated followed by RT-PCR. Relative expression level of indicated genes was detected by real-time PCR. The ATP8B2, EXT2 and OAS1 genes are located next to a CTCF boundary flanking a H3K27me3 domain. The DUSP16, RPLPO, COX6A1 and Actin genes have a CTS, but in the absence of a H3K27me3 domain. All values are normalized to GAPDH. The housekeeping gene UBC has no CTS and served as a negative control. (B) ChIP was performed with antibodies against CTCF, H3K27me3 and H3. For H3K27me3 values are expressed relative to H3 precipitation, CTCF is depicted as percent of input. Arrows indicate primer pairs and their distance to the transcription start site of three analysed genes.

To characterize domain boundaries in more detail, we searched the human genome for typical domain boundaries. According to published results, CTCF is enriched at chromatin domain boundaries separating repressed H3K27me3 marked domains from active domains (10). In order to test the effect of CTCF at the boundary, we searched for such an arrangement with the active chromatin region containing an active gene. In this way gene activity can be monitored in the presence or absence of CTCF and can be used as a readout for insulator function. We chose three boundary model genes, ATP8B2, EXT2 and OAS1, which are active in HeLa cells. These are located in active chromatin, which is flanked by a stretch of more than 20 kb marked by H3K27me3 (Supplementary Figure S1). In each case, a CTCF site (CTS) separates both domains, with a distance of the gene promoter between 6 kb (OAS1) and 0.1 kb (EXT2). For comparison and control we selected four other genes with a similar arrangement of a CTS relative to the promoter of an active gene, but in the absence of any H3K27me3 domain within a 50 kb region. These were DUSP16, RPLPO, COX6A1 and Actin (Supplementary Figure S1). First, we tested whether the expression of these genes is modulated by depleting CTCF from the cells. The protein level of CTCF is decreased upon treatment with specific siRNA (Supplementary Figure S2). As a positive control for CTCF depletion we also determined the amount of CTCF RNA and, as negative control, we tested the expression of the UBC gene. The siRNA-mediated CTCF knockdown resulted in reduced expression of CTCF as well as of the boundary monitoring genes ATP8B2, EXT2 and OAS, as determined by quantitative reverse transcriptase-polymerase chain reaction (RT-PCR) (Figure 1A). In contrast, the genes distant from any H3K27me3 domain boundary (DUSP16, RPLPO, COX6A1 and Actin), while harbouring a CTS in the vicinity of the promoter, did not respond to CTCF depletion, nor did the CTCF negative gene UBC. This suggests that depletion of CTCF from the boundary may result in a spreading of H3K27me3 into the active domain and thereby cause gene repression. We tested this hypothesis using ChIP. First, we verified the presence of CTCF at the boundary with an antibody directed against CTCF. Clearly, CTCF was found at the CTS of all three boundary model genes (Figure 1B). We also verified the chromatin status using an antibody against H3K27me3. The ChIP experiments showed the presence of H3K27me3 in the inactive domain, whereas the CTCF site and the active domain were almost devoid of this histone mark. Besides changes in gene expression induced by CTCF depletion, the crucial test for a CTCF boundary function is to detect changes in chromatin modification. ChIP analysis revealed that CTCF was indeed depleted from the CTS, and that the previously active domain showed an increase in H3K27me3. The strongest increase of this methylation was seen at the CTS itself (Figure 1B). Thus, CTCF depletion induces a loss of gene activity as well as an increase of H3K27me3.

This supported previous results that the loss of CTCF causes spreading of H3K27me3 into an active chromatin domain and thereby reduces the activity of genes located within the active domain. Most importantly, the CTCF site itself was devoid of the H3K27me3 mark, which was strongly re-established after CTCF depletion. This poses the question of whether H3K27me3 demethylation at the CTCF occupied boundary is the cause for or the consequence of CTCF binding.

### CTCF, but not CTCFL, induces a dramatic expansion of a chromatin domain, which is not caused by an activation function of CTCF

To analyse whether demethylation of H3K27me3 is a requirement for CTCF binding at domain boundaries, or whether CTCF causes such a demethylation event, we wanted to target CTCF to a genomic site that is highly marked by H3K27me3. As CTCF binding cannot be experimentally controlled, we fused CTCF to a DNA binding domain specific for the LacO DNA sequence that is not found in eukaryotic genomes. This was achieved by using the LacI DNA binding domain and hundreds of LacO repeats integrated into single genomic loci (28). Specifically, we used the F42B8 cell clone of U2OS cells with a LacO repeat cluster integrated close to the centromere, which constitutes a heterochromatic domain (29). Generating a LacI fusion with any other factor allows for recruiting this factor to the LacO array independently of the hetero- or euchromatic nature of the repeat cluster (29–31) (Supplementary Figure S3).

**Figure 2.**
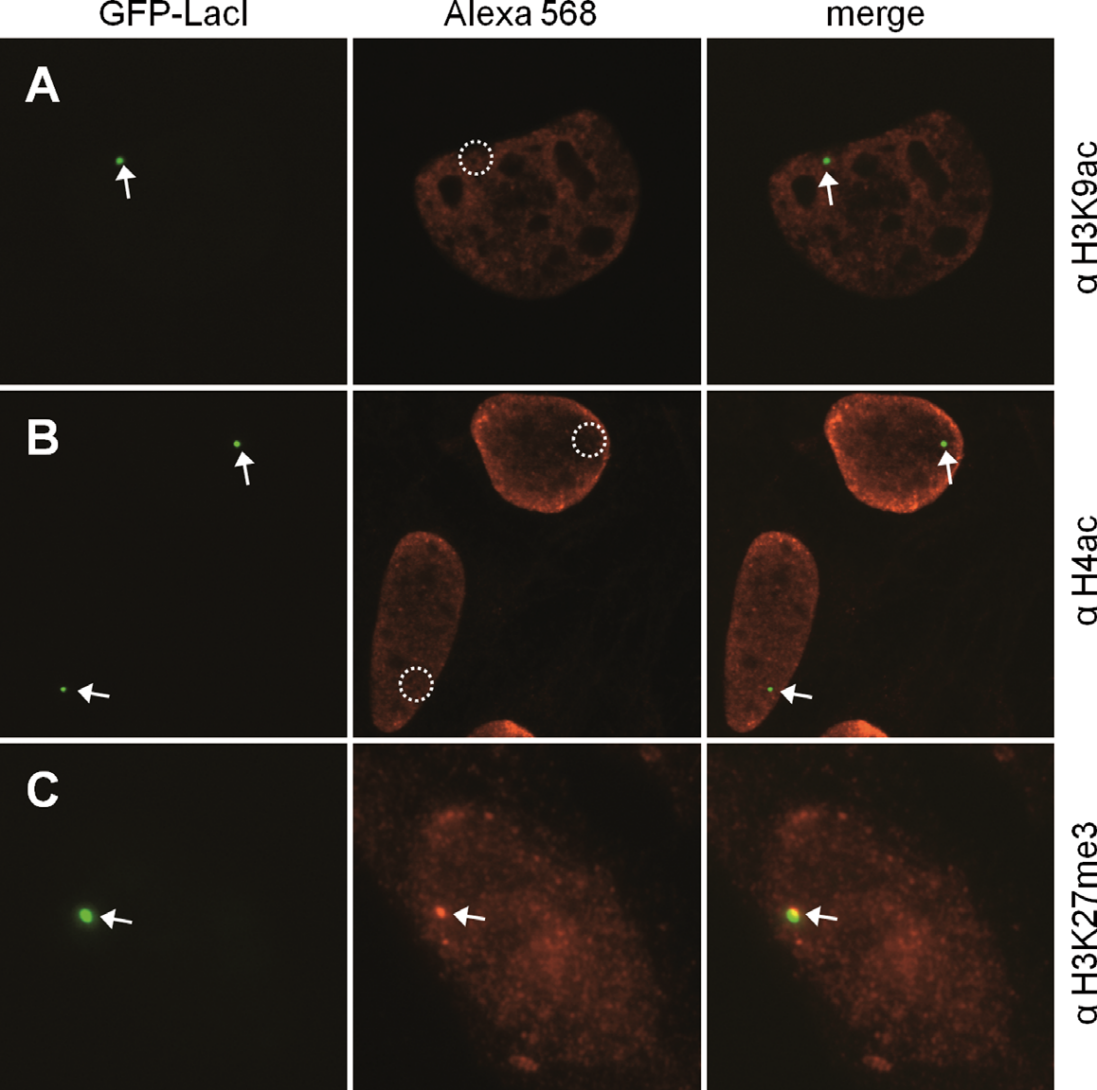
The LacO array of U2OS-F42B8 is positive for H3K27me3. F42B8 cells were transfected with GFP-LacI and incubated for 48 h. Fixed cells were treated with antibodies against (A) H3K9ac, (B) H4ac and (C) H3K27me3. The left panels show the GFP-tagged LacI, the middle show staining of indicated histone modification by indirect immunofluorescence, the third show the merge. Arrows point at positive signals, whereas circles represent lack of a signal.

**Figure 3.**
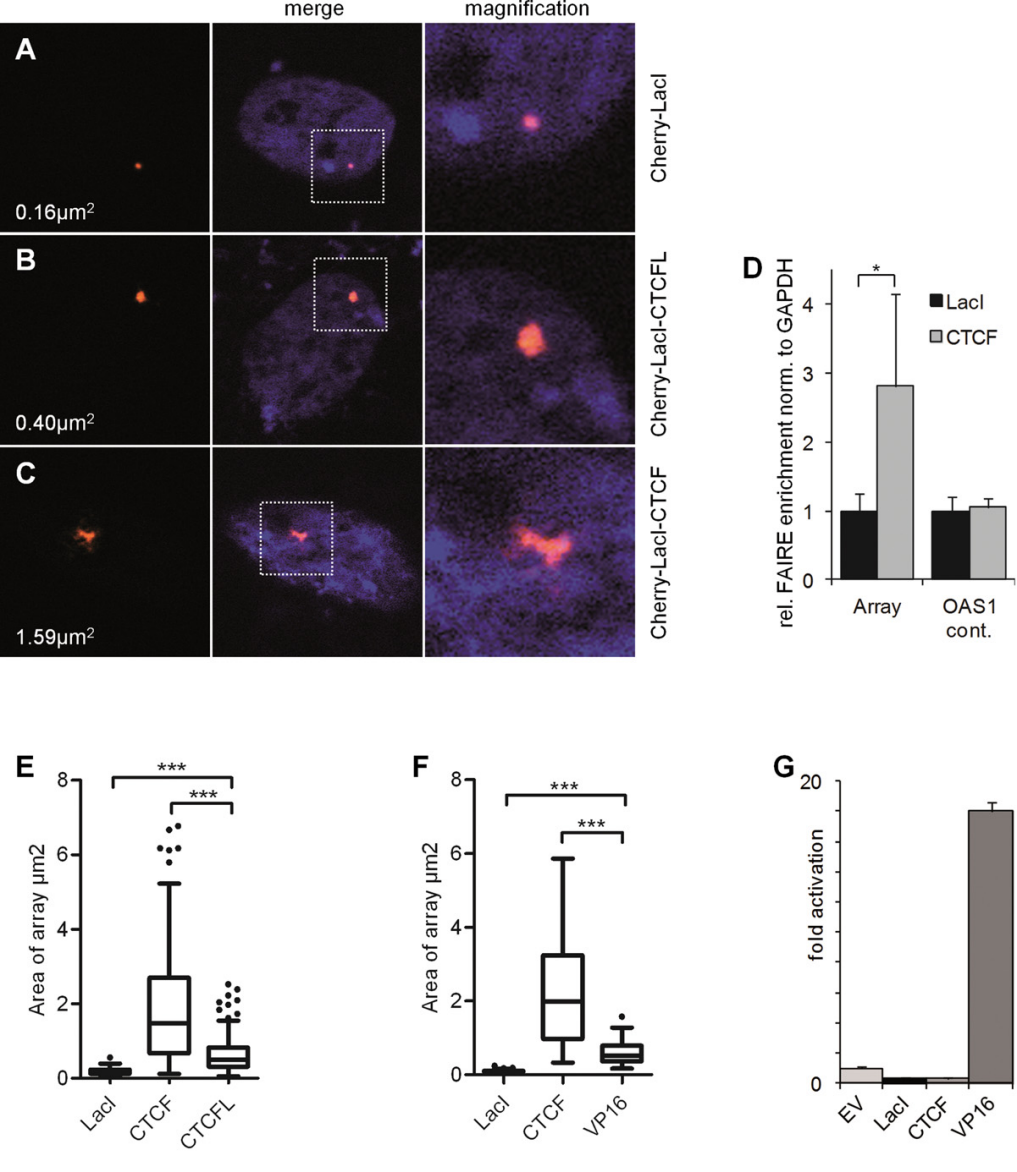
CTCF, but not CTCFL causes a dramatic decondensation of heterochromatin. F42B8 cells were transfected with (A) Cherry-LacI, (B) Cherry-LacI-CTCFL and (C) Cherry-LacI-CTCF. The cells were fixed after 48 h and stained with Hoechst to visualize the nucleus. The size (in μm^2^) of representative arrays was measured by the Volocity software. The left panels show the Cherry-tagged proteins, the middle show the merge with Hoechst and the right is a magnification of the merge. (D) FAIRE assay was performed on F42B8 cells transfected with either LacI or LacI-CTCF (CTCF). Fold change of DNA enrichment of Lac array sequences and of a CTCF negative control site 8 kb downstream of the OAS1 CTCF site (neg. contr.) was determined and expressed relative to the FAIRE signal of the GAPDH promoter sequence. The GAPDH control gene does not respond to LacI-CTCF expression. (E) The size of ∼100 arrays was measured and presented as a box plot, with whiskers as defined according to Tukey. Significance was controlled with a two-tailed Mann–Whitney test. (F) Same as in E but CTCFL was replaced by VP16 and Cherry by GFP. (G) 293T cells were transiently co-transfected with a plasmid containing 7 LacI binding sites in front of the luciferase reporter and the indicated vectors. All values were normalized to LacZ. EV, empty vector; LacI, GFP-LacI; CTCF, GFP-LacI-CTCF; VP16, GFP-LacI-VP16.

Using a LacI fusion with the green fluorescent protein (GFP), the location of the repeat array could easily be determined at the cytological level. Using antibodies directed against active (euchromatic) histone modifications, such as H3K9ac, H4ac, no signal of the GFP marked array was detectable after immunostaining (Figure 2A and B). In contrast, antibodies against the repressive (heterochromatic) mark H3K27me3 easily identified the GFP marked spot (Figure 2C). To analyse the effect of CTCF on this chromatin domain, we generated a GFP-LacI-CTCF expression vector. Also, to increase the combinatorial use of expression vectors, we generated similar fusions with the mCherry-coding region replacing GFP. Expression of Cherry-LacI clearly targets the fusion protein to a localized structure within the nucleus (Figure 3A). Targeting was sensitive to treatment with IPTG (Supplementary Figure S4), a substance known to interfere with DNA binding of the LacI repressor. Expression of Cherry-LacI-CTCF caused an increase in the labelled nuclear structure (Figure 3). Cherry-LacI co-localizes with GFP-LacI-CTCF (Supplementary Figure S5), allowing the conclusion that the structure seen in Figure 3 is the LacO array, which is strongly enlarged when compared to the Cherry-LacI or GFP-LacI bound array. We quantified the LacI-CTCF induced expansion of the array and found that in more than 80% of the transfected cells such an array enlargement could be observed (Supplementary Figure S6). In order to verify whether this expansion is caused by chromatin opening of the heterochromatic array we carried out a FAIRE assay (32). Expression of GFP-LacI-CTCF causes a 3-fold increase in solubilized array DNA as compared to GFP-LacI expression. A CTS negative control site in the genome did not respond to CTCF expression (Figure 3D). In order to quantify the chromatin opening activity we measured the size of the array within the micrographs using the Volocity software. We measured a 10- to 20-fold increase of the 2D area, when expressing LacI-CTCF (Figure 3E and F). Thus, we conclude that in contrast to LacI, the LacI-CTCF factor is actively opening a heterochromatic region inserted in the genome.

One could envisage that the opening of repressed and compact chromatin might be mediated by the property of CTCF to activate genes (30). To address this aspect we fused the well-characterized activation domain of VP16 from Herpes simplex virus (33) with the LacI DNA binding domain. In a transient reporter assay, using a Luciferase gene controlled by 7 LacO sequences, the LacI-VP16 construct resulted in a more than a 100-fold activation of the reporter, in contrast to LacI-CTCF that showed a marginal repression (Figure 3G). Expression of GFP-LacI-VP16 within the F42B8 cells caused an expansion of the LacO array of about 6-fold, which is about 4-fold lower as compared to the CTCF-induced expansion (Figure 3F). In other words, it is not the activation function of CTCF that is causing the array expansion, since the strong activator VP16 is significantly less efficient in enlarging the LacO domain.

CTCFL, the paralogous factor of CTCF, is expressed in germ cells and in some tumour types (34–37). CTCF and CTCFL are co-expressed in pathological cases, as well as in the testes and to some extent in other tissues as well (38). Since the DNA binding domain of both factors is very similar, a potential competitive function has been proposed (36). Therefore, we wanted to determine whether both factors differ in chromatin expansion activity. We generated LacI-CTCFL fusion constructs similar to LacI-CTCF. Clearly, LacI-CTCFL induced an array enlargement (Figure 3B) in a comparable percentage of cells as seen with LacI-CTCF. However, when measuring the size of the expanded array a substantial difference was observed, such that LacI-CTCFL was about 3-fold less efficient (Figure 3E) than CTCF. This suggests that more than one domain may cause chromatin opening by CTCF, with a low level opening shared by CTCF and CTCFL, whereas the full opening activity is specific for CTCF. This aspect is addressed below.

### CTCF-induced chromatin opening is mediated by the N-terminus plus zinc finger domain and involves H3K27me3 demethylation

**Figure 4.**
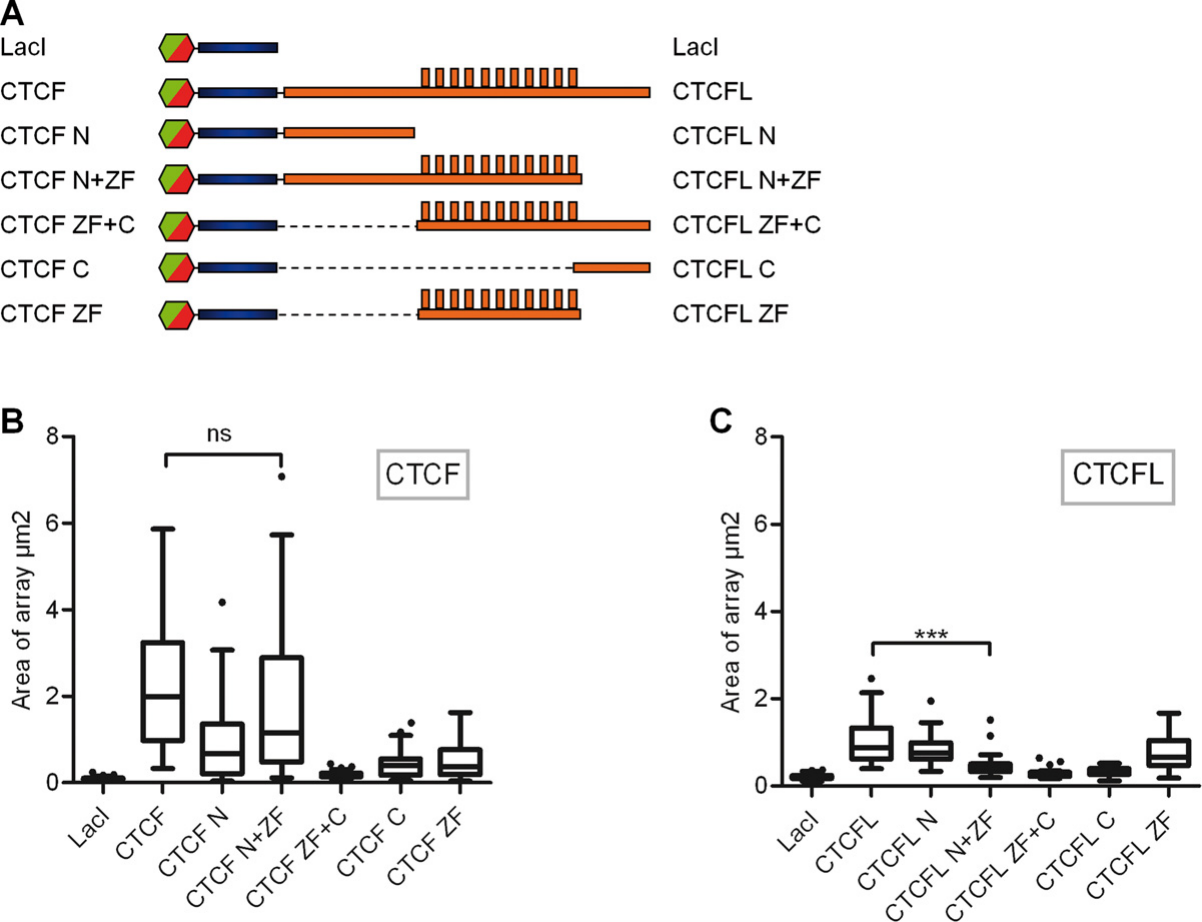
CTCF N-terminus plus Zinc finger harbour the strong, CTCF-specific decondensation domain. (A) Schematic representation of CTCF and CTCFL full-length and deletion constructs. F42B8 cells were transfected with either CTCF (B) or CTCFL (C) constructs as indicated and incubated for 48 h. The size of ∼50 arrays was determined and presented as box plots (see legend to Figure 3D).

To identify protein domains that are required for chromatin expansion we generated a set of deletions of CTCF and CTCFL fused to LacI in combination with either GFP or Cherry. These deletions were designed such that the three domains of CTCF and CTCFL, the N-terminus, the central zinc finger region or the C-terminus, were deleted or retained within the constructs (Figure 4A). All of these were transfected into the F42B8 cells and the size of the array was determined. As seen above, full-length CTCF showed the strongest expansion activity, when compared to full-length CTCFL (Figure 4B and C). Deleting the C-terminus of CTCF (LacI-CTCF-N+ZF) did not significantly reduce this activity, whereas all other CTCF constructs caused a substantial reduction in the opening of the chromatin array.

Thus, the comparison between CTCF and CTCFL domains revealed that only CTCF harbours the powerful chromatin opening domain within the N-terminus plus zinc finger region.

**Figure 5.**
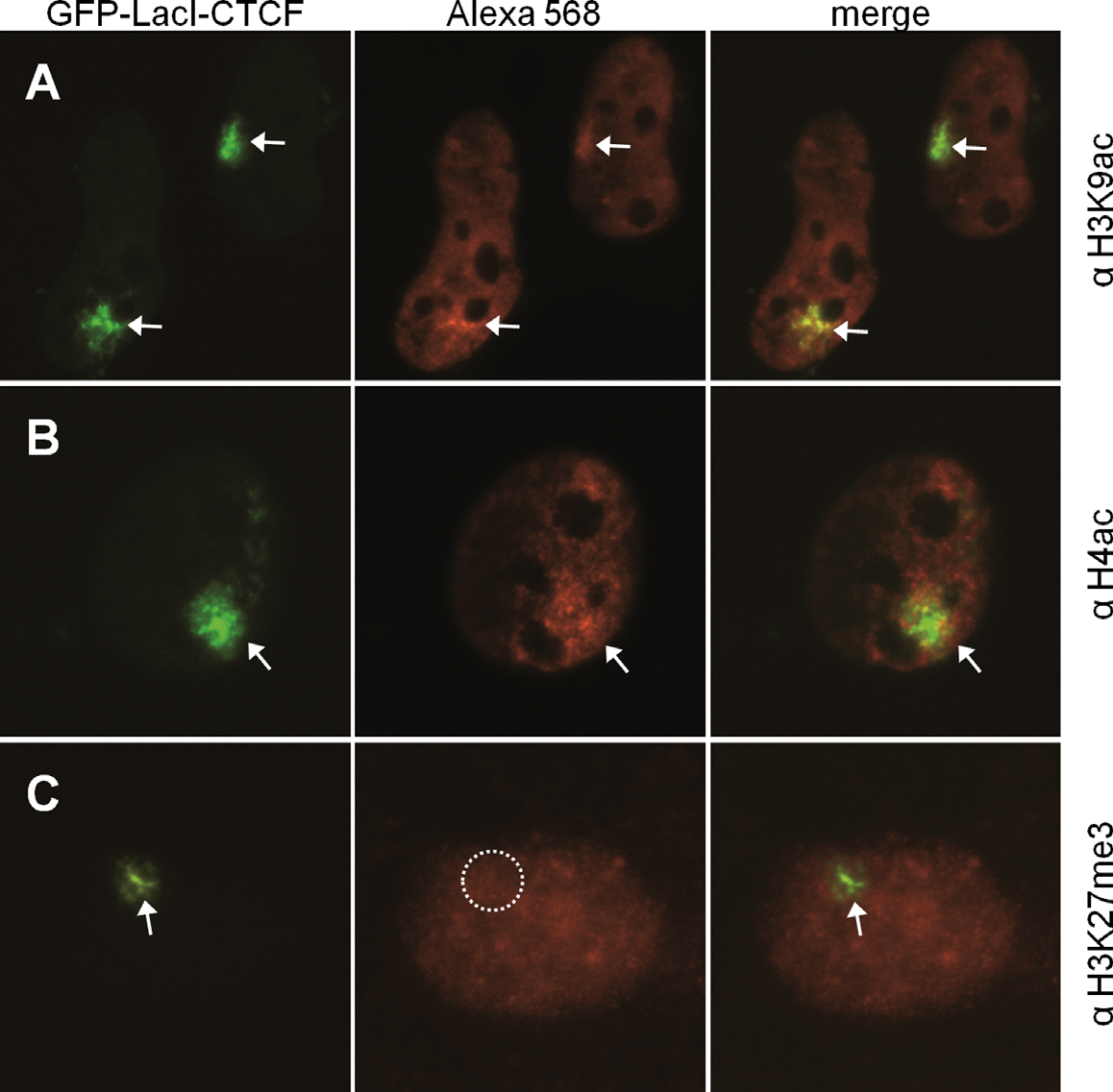
CTCF converts repressed chromatin into active chromatin. F42B8 cells were transfected with GFP-LacI-CTCF and incubated for 48 h. Fixed cells were treated with antibodies against (A) H3K9ac, (B) H4ac and (C) H3K27me3. The left panels show the GFP-tagged LacI-CTCF, the middle show staining of indicated histone modification by indirect immunofluorescence, the third shows the merge. Arrows point at positive signals, whereas circles represent lack of a signal.

Chromatin opening is usually associated with, or is caused by, histone modifications. Since we found the closed array of the F42B8 cells to be devoid of active chromatin marks, showing rather the repressed chromatin modification of H3K27me3, we wanted to know whether LacI-CTCF induces changes in histone modification. After expression of GFP-LacI-CTCF we tested for the presence of the same chromatin marks as identified after LacI-GFP transfection in Figure 2. In contrast to the GFP-LacI transfection, GFP-marked chromatin domains showed positive staining after GFP-LacI-CTCF transfection of F42B8 cells for H3K9ac and H4ac (Figure 5A and B). Furthermore, the repressive H3K27me3 mark was no longer detected (Figure 5C). To generalize this finding we used another cell clone (F6B8 cells) with three separate integration sites of the LacO cluster (29). Upon transfection with GFP-LacI or GFP-LacI-CTCF again, we observed a loss of H3K27me3 accompanied by an expansion of the arrays (Supplementary Figure S7). In this experimental setting the transfected cells were kept for 2 days, a time period sufficient for two cell cycles. At this point, we did not know whether the removal of the H3K27me3 mark is dependent on S phase DNA replication, or whether active removal of this mark is seen in arrested cells as well. Therefore, we repeated the experiment with arrested cells. In order to do so and to allow for the expression of GFP-LacI-CTCF we made use of IPTG to prevent DNA binding of CTCF. Upon cell-cycle arrest and removal of IPTG we measured the size of the array, as well as the staining intensity of an antibody against H3K27me3 (Supplementary Figure S8). Again, chromatin opening and removal of H3K27me3 were observed.

Thus, at a heterochromatic binding site CTCF is actively opening the compact array structure and removing the H3K27me3 modification independent of the cell cycle.

### Chromatin expansion is preceded by a CTCF-induced removal of the H3K27me3 mark

**Figure 6.**
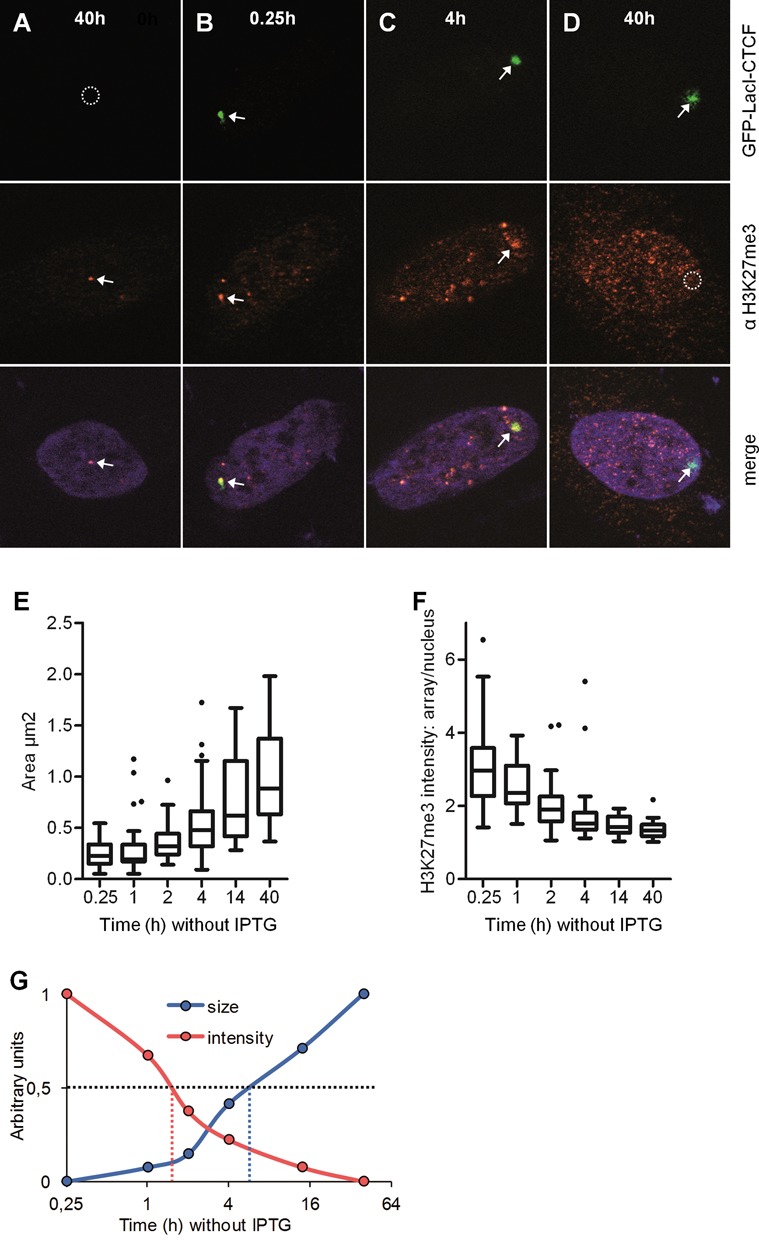
CTCF-induced decondensation and demethylation of the array occur very rapidly. F42B8 cells were transfected with GFP-LacI-CTCF in the presence of 150 μM IPTG and incubated for 12 h. IPTG was removed from the medium for indicated time periods (A–D) followed by treatment with 4% PFA. Immunostaining was performed with an antibody against H3K27me3 and the nucleus was stained with Hoechst. In (A) an untransfected cell is shown. Arrows point at positive signals, whereas circles indicate the lack of a signal. (E) The size of ∼50 arrays was measured using the Volocity software and shown as a box plot. (F) The H3K27me3 value was detected as the ratio of signal intensity of the array to signal intensity of the whole nucleus defined by Hoechst staining. (G) The data sets of the size and the signal intensity are plotted as arbitrary units, whereas the mean values are set as 0 (lowest) and 1 (highest), respectively.

To determine the sequence of the molecular events of H3K27me3 removal and of chromatin domain opening we needed to determine the time required to establish these changes. We could not simply use different time points after transfection to do so, since expression of the fusion proteins takes time and has to be distinguished from the time after DNA binding. Therefore, again, we made use of the IPTG substance to interfere with DNA binding of the GFP-LacI-CTCF fusion. Removal of IPTG allows for DNA binding and, therefore, the time after DNA-binding required to induce the molecular changes at the chromatin array can be determined. The size of the LacO array did not change significantly immediately after IPTG removal and for up to 2 h of GFP-LacI-CTCF binding (Figure 6B an E). However, at 4 h the array size started to increase and continued to do so up to 40 h (Figure 6C–E). Longer incubations did not further increase the size of the array (not shown), and as such the major changes in domain opening are observed between 4 and 40 h of LacO binding of CTCF.

A similar analysis was done for the presence of the H3K27me3 mark. After transfection with GFP-LacI-CTCF and IPTG removal at the different time points, cells were fixed and stained with the antibody specific for H3K27me3. We measured the intensity of immunofluorescence staining and controlled for staining variation between nuclei by determining the overall staining intensity for the total nuclear area with the help of the Volocity software. The staining intensity over the array area was determined and was then divided by the overall nuclear intensity (Figure 6B–D and F). The strongest change in H3K27me3 staining intensity is observed within the first 4 h. Time periods of GFP-LacI-CTCF binding longer than 4 h resulted only in a marginal decrease in antibody staining. When comparing these results with the time kinetic determined for array expansion (Figure 6E), two phases are evident. Within the first 4 h a strong reduction in H3K27me3 is observed, whereas the dramatic chromatin expansion is visible only after 4 h. To better compare and to visualize the changes, we plotted the measured changes as arbitrary units from 0 (average of minimum size and minimum antibody staining) to 1 (average of maximum size and maximum antibody staining). Again, the half maximum change of antibody staining occurs before 1.5 h, whereas the half maximum change of the size increase is found after 6 h (Figure 6G).

**Figure 7.**
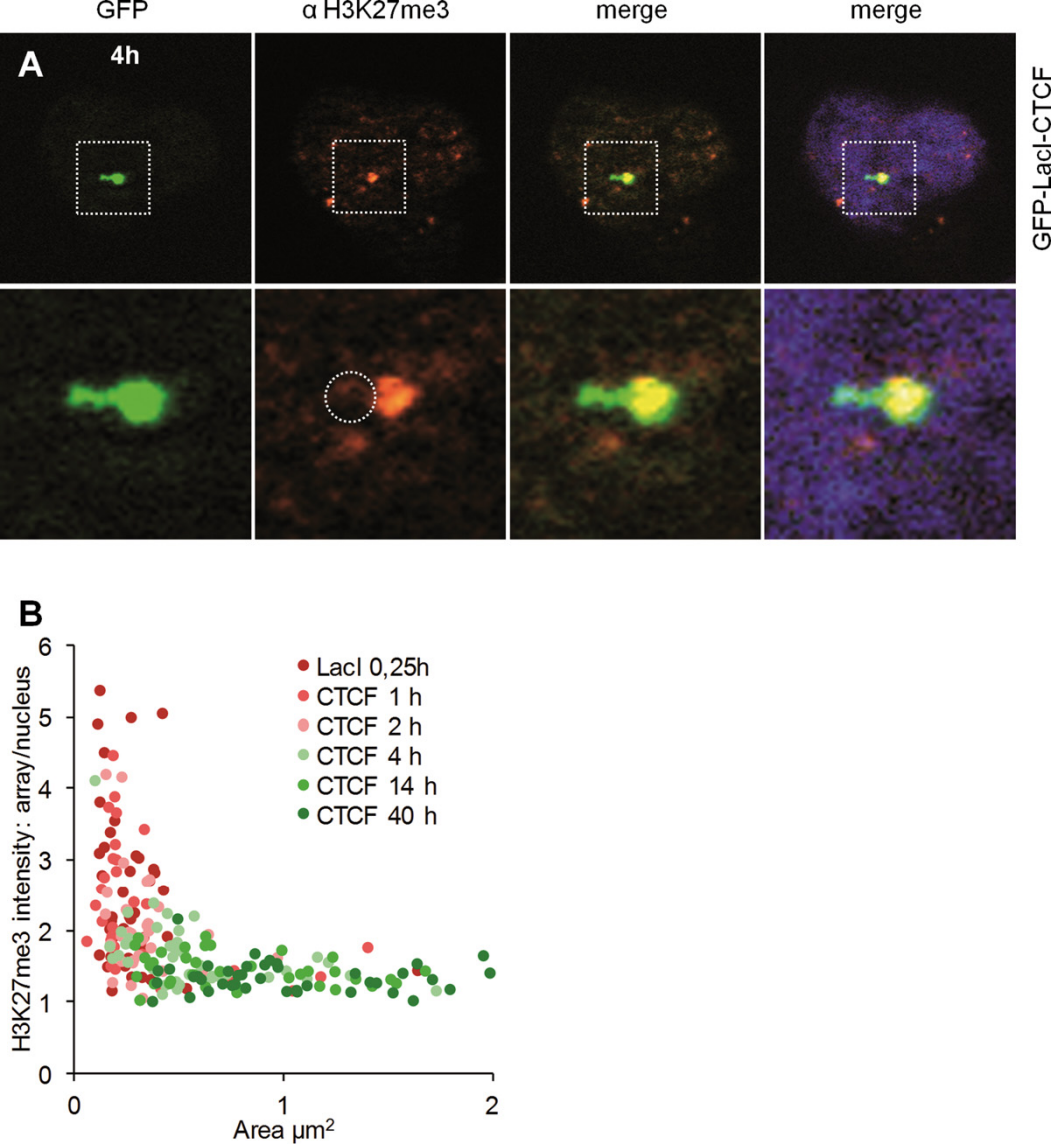
CTCF-induced demethylation of H3K27 is completed before the chromatin is fully expanded. F42B8 cells were transfected with GFP-LacI-CTCF in the presence of 150 μM IPTG and incubated for 12 h. IPTG was removed from the medium for the indicated time periods and fixed with 4% PFA. Immunostaining was performed with an antibody against H3K27me3, DNA was stained with Hoechst. (A) A representative picture after 4 h of IPTG removal is presented. The left panels show the GFP-tagged LacI-CTCF, the second show staining of H3K27me3 by immunofluorescence, the third and fourth show the merge. Squares define the magnified area, and the circle indicates the lack of a H3K27me3 signal over the expanded chromatin region. (B) Signal intensities of arrays were plotted according to their size and time of IPTG removal.

Such a difference in demethylation and domain expansion should also be detectable at individual array domains when analysed after short time periods (2–4 h) of IPTG removal and DNA binding. Microscopic inspection revealed that expanded regions are negative for H3K27me3 within array areas as well, whereas a more compact region is still positive (Figure 7A). In order to further analyse histone demethylation in comparison to chromatin array expansion, we simultaneously determined H3K27me3 staining and the size of the array, and plotted the H3K27me3 intensity against the size of the array for each measurement (Figure 7B). Clearly, the strongest changes in histone demethylation are seen at the state of arrays that are smaller than 0.5 μm^2^. These are heavily enriched for short time periods of DNA binding at 2 h or shorter (reddish coloured points). In contrast, within larger arrays (between 0.5 and 2 μm^2^) the H3K27me3 staining is already at its minimum. These points are clearly enriched for longer periods of DNA binding of 4 h and longer. These measurements (Figures 6 and 7) all confirm that CTCF not only induces the removal of H3K27me3, but furthermore, that this occurs before chromatin expansion.

### CTCF-induced removal of the H3K27me3 mark is accompanied by a transient H3.3 incorporation

**Figure 8.**
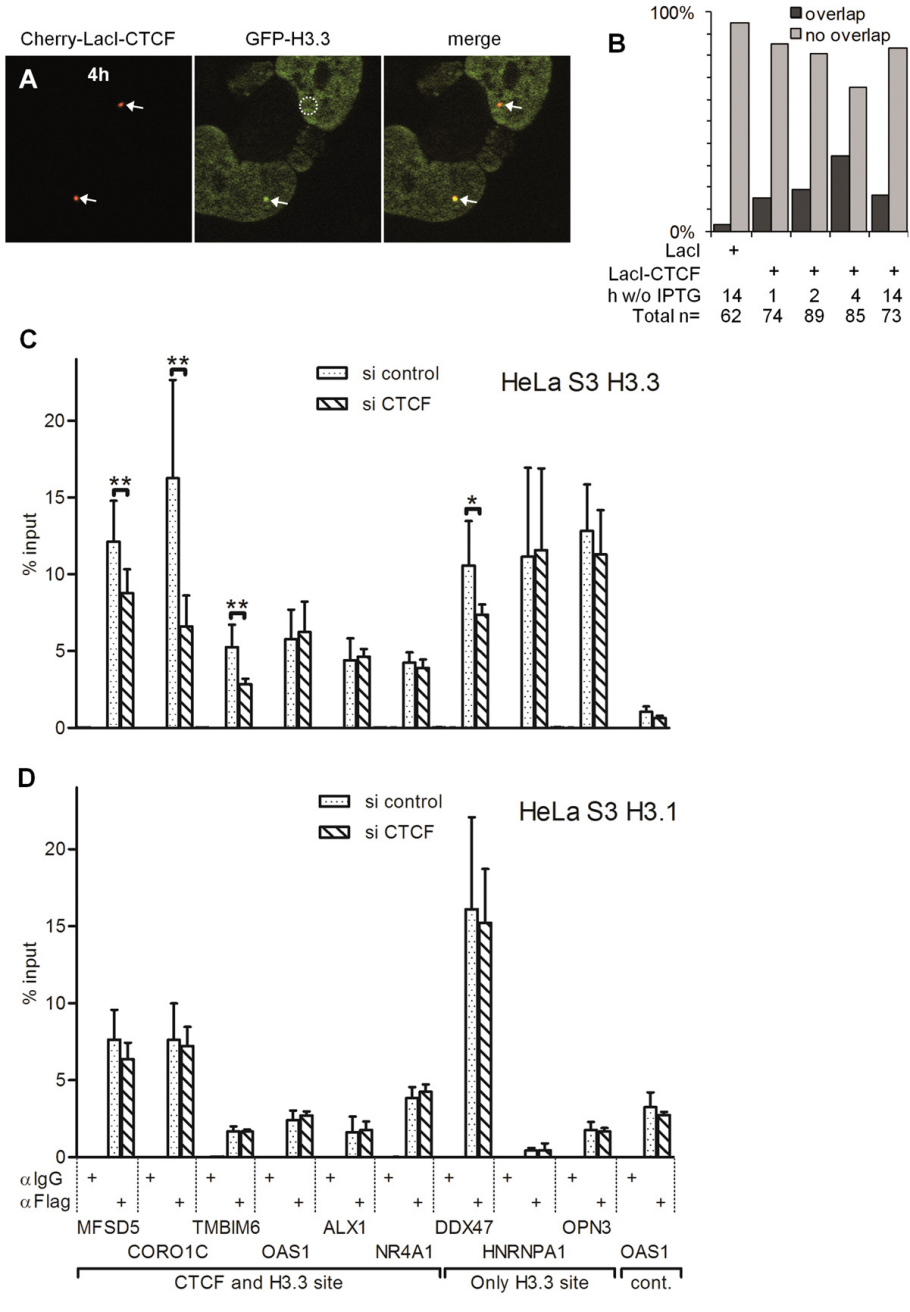
CTCF-induced demethylation of H3K27me3 is accompanied by incorporation of histone variant H3.3. F42B8 cells were co-transfected with either Cherry-LacI or Cherry-LacI-CTCF and GFP-H3.3 in the presence of 150 μM IPTG and incubated for 24 h. IPTG was removed from the medium for the indicated time periods. (A) A representative picture after 4 h of IPTG removal is shown. Arrows point at positive signals, whereas the circle indicates lack of a signal. (B) Indicated number of arrays were analysed for an overlap with GFP-H3.3. NChIP-qPCR of Flag or control immunoglobulin G (IgG) in HeLa H3.3-Flag-HA (C) or HeLa H3.1-Flag-HA (D) cells. Indicated loci tested for H3.3 binding after control or CTCF siRNA treatment. *P*-values were calculated by paired two-tailed Student's *t*-test (*n* = 6) and asterisks represent values from 0.05 to 0.01(*) and 0.01 to 0.001 (**), respectively.

In search for a mechanism of H3K27me3 demethylation we tested the histone demethylases UTX (39) and JMJD3 (40) in addition to inhibitory substances known to interfere with histone demethylation (SAHA (41); Methylstat (42)), but without any substantial effect (data not shown). These results and the cell-cycle arrest experiment (above, Supplementary Figure S8) suggest that the modified histones might be replaced in an S phase independent manner. The histone variant H3.3 has been found to be incorporated into chromatin independent of DNA synthesis (43). Furthermore, it has been shown that H3.3 is significantly enriched at regions reduced in nucleosomal occupancy, such as active promoters, enhancers or insulator elements. This is further correlated with CTCF binding sites (26). Therefore, we tested whether the exchange of methylated H3 with H3.3 might be a potential mechanism for removing the methylation mark. Such a mechanism requires that upon CTCF binding H3.3 will be incorporated into chromatin. We tested this by co-expression of Cherry-LacI-CTCF and GFP-H3.3 in the LacO cell line. This resulted in a co-localization of GFP-H3.3 at the Cherry-LacI-CTCF bound array (Figure 8A). As the number of co-localizing events varied between experiments, we predicted that the H3.3 incorporation might occur transiently. Therefore, we used the IPTG incubation to allow for controlled time points of DNA binding after IPTG removal. Counting the number of cases with an overlap of the Cherry-LacI-CTCF mark with the GFP-H3.3 label at different time points revealed a peak of more than 30% of overlapping cases at 4 h of DNA-binding, with a half-maximum between 1 and 2 h. This transient H3.3 incorporation is CTCF dependent, since Cherry-LacI expression does not cause GFP-H3.3 to be incorporated into the LacO chromatin.

Thus, it can be concluded that the rapid removal of the H3K27me3 mark, which precedes chromatin opening, occurs concomitantly to the loss of H3K27me3.

To further monitor the CTCF-dependent incorporation of the histone variant H3.3 *in vivo*, we made use of a human HeLa S3 cell clone stably expressing H3.3-Flag-HA (44). These cells were transfected with siRNA against CTCF, which resulted in CTCF depletion at protein and RNA levels (Supplementary Figure S9). We analysed regions of H3.3 nucleosomes with and without an overlapping CTCF binding site. Genome-wide CTCF binding data in HeLa S3 cells generated by the ENCODE project were compared with ChIP-seq profiles of H3.3 (45). We then chose six regions enriched for CTCF and H3.3, three that were only enriched for H3.3, and one that showed no signal for either CTCF or H3.3 as a control site (Figure 8C). The presence of CTCF was verified at these sites (Supplementary Figure S10). Depletion of CTCF showed a highly significant reduction in H3.3 at the sites MFSD5, CORO1C and TMBIM6 as compared to cells treated with unspecific siRNA (Figure 8C). The OAS1, ALX1 and NR4A1 sites displayed no change in H3.3 levels. We did not expect massive changes or that all CTCF sites respond with reduced H3.3 incorporation since the experimental design only allows testing H3.3 at sites permanently bound by CTCF (45), whereas the LacO targeting experiment suggests a transient H3.3 recruitment.

Sites devoid of CTCF did not change in terms of H3.3 content upon CTCF depletion (HNRNPA1 and OPN3), or showed a less significant change (DDX47). Also, the negative control site OAS1 did not respond to the depletion of CTCF. To further underline the above findings we performed the same experiments with a HeLa S3 H3.1-Flag-HA cell clone. Depletion of CTCF protein in these cells resulted in no significant changes of H3.1 level at all genomic regions tested (Figure 8D), although CTCF binding was significantly reduced at all CTCF sites in S3 H3.3 and S3 H3.1 (Supplementary Figure S10).

**Figure 9.**
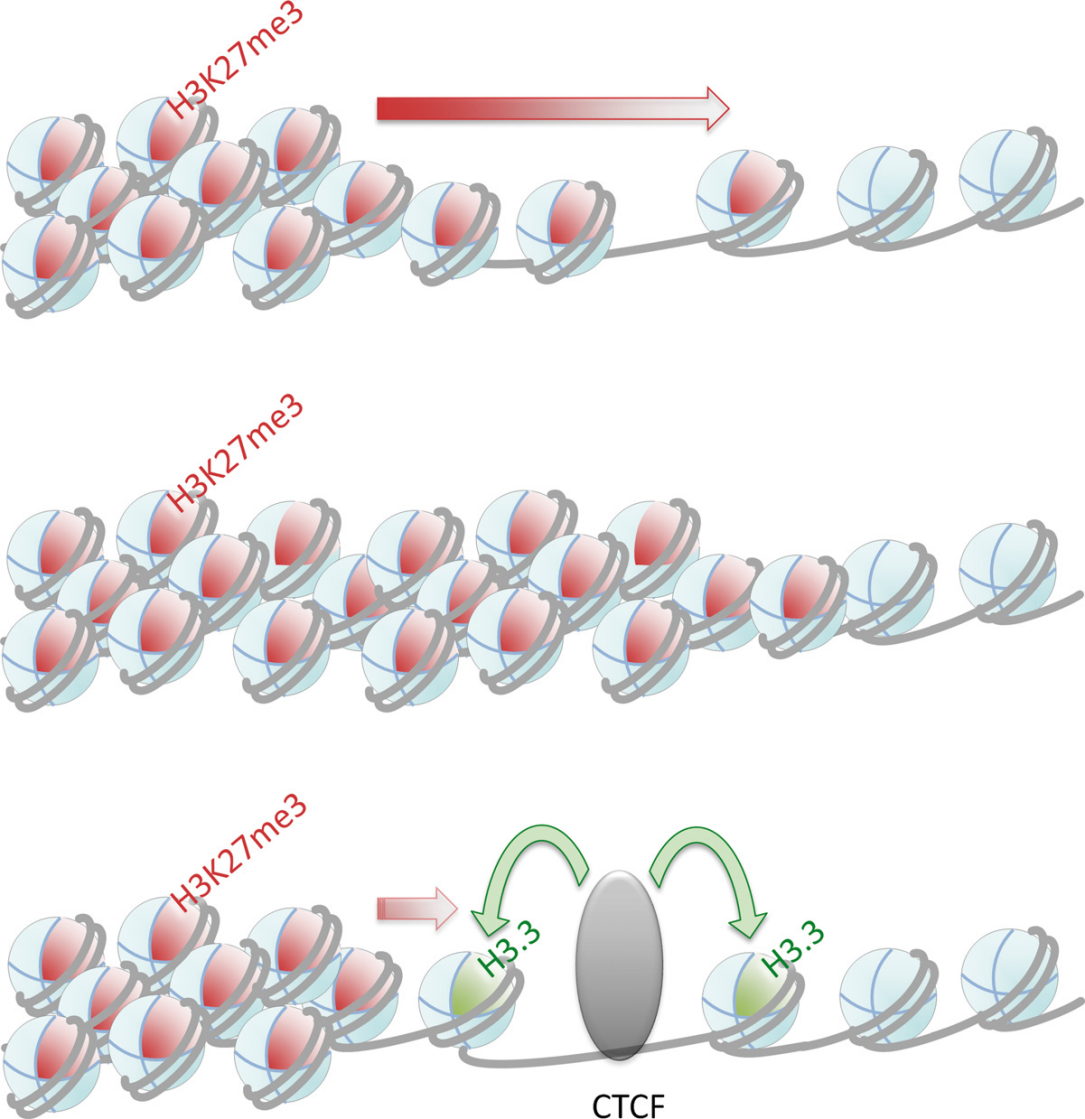
Insulator action. Top: In the absence of the insulator factor CTCF a repressive chromatin modification, such as H3K27me3 (red octant-sphere) within nucleosomes (grey sphere), shows the tendency to spread from a repressed chromatin domain into an active chromatin domain devoid of this mark. Bottom: In the presence of CTCF, the variant histone H3.3 (green octant-sphere) replaces modified histone H3. H3.3, often in combination with H2A.Z, destabilizes nucleosomes, which may have to be continuously replaced. Such an activity would act antagonistically to a spreading of the H3K27me3 mark.

Together these results suggest that the rapid removal of the H3K27me3 mark, which precedes chromatin opening as tested with the LacO array model, is at least in part mediated by the transient incorporation of H3.3. This is supported by the analysis of endogenous CTCF/H3.3 sites, which show a reduced H3.3 incorporation upon CTCF depletion. Thus, it can be envisaged (Figure 9) that CTCF incorporates H3.3, which causes depletion of stable nucleosomes and thereby interferes with spreading of the repressive marks into the active domain.

## DISCUSSION

From early on it has been known that chromatin binding sites for the insulator factor, CTCF, show a general depletion of nucleosomes with the flanking and remaining nucleosomes being marked by active histone marks (for reviews see (6,46)). It has been postulated that such active marks prevent the spreading of repressive marks, such as H3K27me3, from one side of the insulator to the other side (10,47). Upon CTCF depletion, the empty binding site usually increases in nucleosomal density and gains repressive histone marks. This is usually interpreted that in the absence of the insulator protein CTCF the repressive histone marks are spreading into the previously active chromatin domain (46). That this is indeed the case is supported by our analysis of three boundary model genes, ATP8B2, EXT2 and OAS1. Upon CTCF depletion, the three genes are repressed in activity and the previously active chromatin accumulates the H3K27me3 mark. Furthermore, H3K27me3 modification at the empty CTCF site dramatically increases to a similar level as in the flanking domain of repressed chromatin. Similar observations have been made at other CTCF binding sites in vertebrates (20) as well as in *Drosophila* (22). A substantial increase in H3K27me3 was shown in Friedreich ataxia patients, who have a severe depletion of CTCF in the 5′ untranslated sequence of the frataxin gene (FXN) (48). These observations and other experiments clearly showed that the presence of CTCF prevents the formation of heterochromatic nucleosomes at the CTS. Nevertheless, previous experiments could not resolve the question of whether CTCF binding requires regions reduced in nucleosomal density and devoid of heterochromatin marks, or whether CTCF is actively modifying its binding site such that the nucleosomal density is lowered and the heterochromatic marks are removed.

As CTCF binding to endogenous sites cannot be experimentally manipulated we used the LacI DNA binding domain, which is known to bind to chromatin irrespective of the heterochromatic state (29–31). With this tool we could compare the chromatin ‘opening’ function of CTCF with the one mediated by CTCFL, the paralogous factor of CTCF (36). The extent of the expansion is specific for the CTCF N-terminal and zinc-finger domain, and is not achieved after expression of the strong activator LacI-VP16 or by LacI-CTCFL. This indicates that the potential unfolding activity of CTCF may allow for binding at closed chromatin sites, which upon binding might be opened. In contrast, CTCFL, which is devoid of a strong chromatin unfolding activity, might need pre-opened and active chromatin as a requirement for binding. This notion is very much supported by the analysis of genome-wide CTCFL binding. When ES cells with and without CTCFL are compared it was shown that CTCFL is not bound to all CTCF sites, rather CTCFL is enriched at active and open chromatin regions (37).

Apparently, CTCF is actively opening heterochromatin, which could potentially be mediated by several molecular changes in histone modification. Opening itself may require other factors, such as the remodelling complex CHD8, which has been shown to interact with CTCF (49). Here we could show that the repressive histone modification H3K27me3 is lost after CTCF binding and that this loss precedes the opening function. H3K27me3 is the hallmark for silent chromatin domains, which when flanked by CTCF are devoid of this mark in the vicinity of the CTCF binding site (10,22,25,50,51). This result indicates that CTCF induces open chromatin and removes the repressive mark H3K27me3. Other barrier sites have been documented as well, such as the composite site at the chicken β-globin locus (17) or sites devoid of CTCF (10–12,18,19). All of these sites have in common a barrier site that is marked by a region depleted of nucleosomes, and a lack of repressive marks in the vicinity. Furthermore, the variant histone H3.3 has been found to be enriched at insulators (26,52,53). The presence of H3.3 and H2A.Z generates unstable nucleosomes, which may be the cause for ‘nucleosome-free regions’ (52). Indeed, at the genome-wide level it has been shown that H3.3 counteracts the association of H1, suggesting that H3.3 helps to keep chromatin in an open conformation (54). This led to the proposal that the presence of H3.3 prepares CTCF sites to be bound by CTCF (27). Our data clearly show that H3.3 incorporation into chromatin is induced by CTCF. This supports a role for H3.3 in insulator function as a consequence of CTCF binding, rather than having a role in CTCF binding to chromatin. Such a functional role could be connected to the instability of H3.3 nucleosomes, as CTCF bound to chromatin may permanently induce H3.3 incorporation, which in turn leads to frequent histone exchanges (Figure 9). This activity very likely will counteract any spreading of repressive chromatin marks through the CTCF bound insulator site.

## SUPPLEMENTARY DATA

Supplementary Data are available at NAR Online.

SUPPLEMENTARY DATA
