# Supplementary material for: CTCF induces histone variant incorporation, erases the H3K27me3 histone mark and opens chromatin
Source: Nucleic Acids Res. 2014 Oct 7;42(19):11941–51. doi: 10.1093/nar/gku937 (PMC4231773; doi:10.1093/nar/gku937)
Supplement: SUPPLEMENTARY DATA [file supp_42_19_11941__index.html]

CTCF induces histone variant incorporation, erases the H3K27me3 histone mark and opens chromatin — SUPPLEMENTARY DATA 

# CTCF induces histone variant incorporation, erases the H3K27me3 histone mark and opens chromatin

## SUPPLEMENTARY DATA

**Files in this Data Supplement:**

- SUPPLEMENTARY DATA
- SUPPLEMENTARY DATA
