# Supplementary material for: CTCF induces histone variant incorporation, erases the H3K27me3 histone mark and opens chromatin
Source: Nucleic Acids Res. 2014 Oct 7;42(19):11941–51. doi: 10.1093/nar/gku937 (PMC4231773; doi:10.1093/nar/gku937)
Supplement: SUPPLEMENTARY DATA [file supp_gku937_nar-03423-x-2013-File012.pdf]

ATP8B2

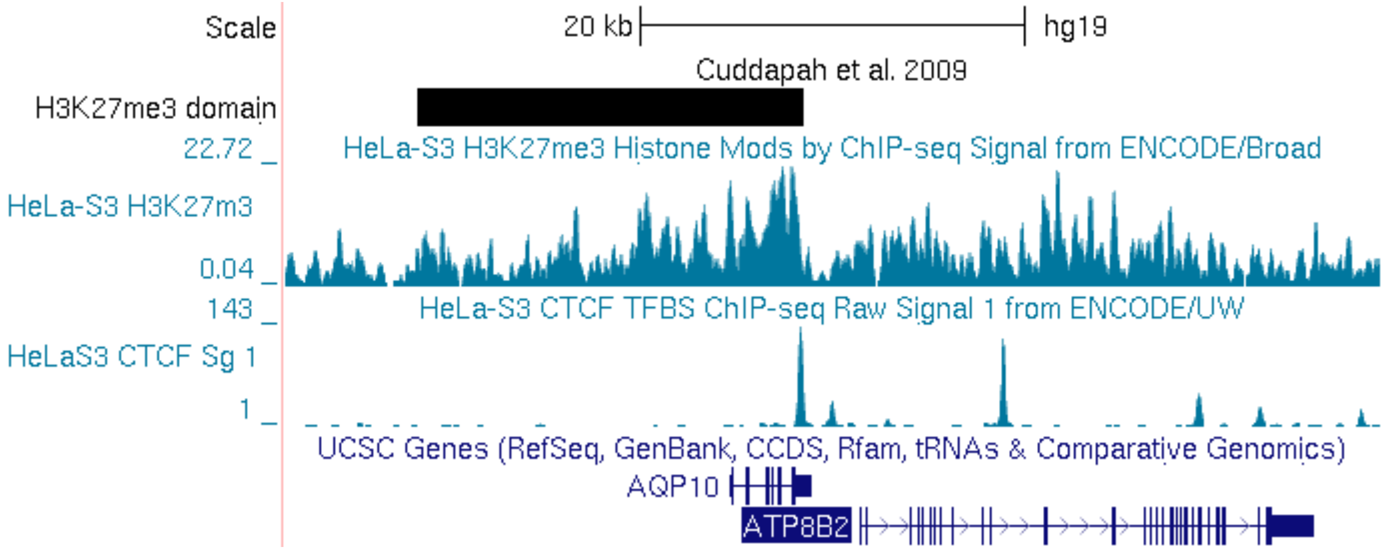

EXT2

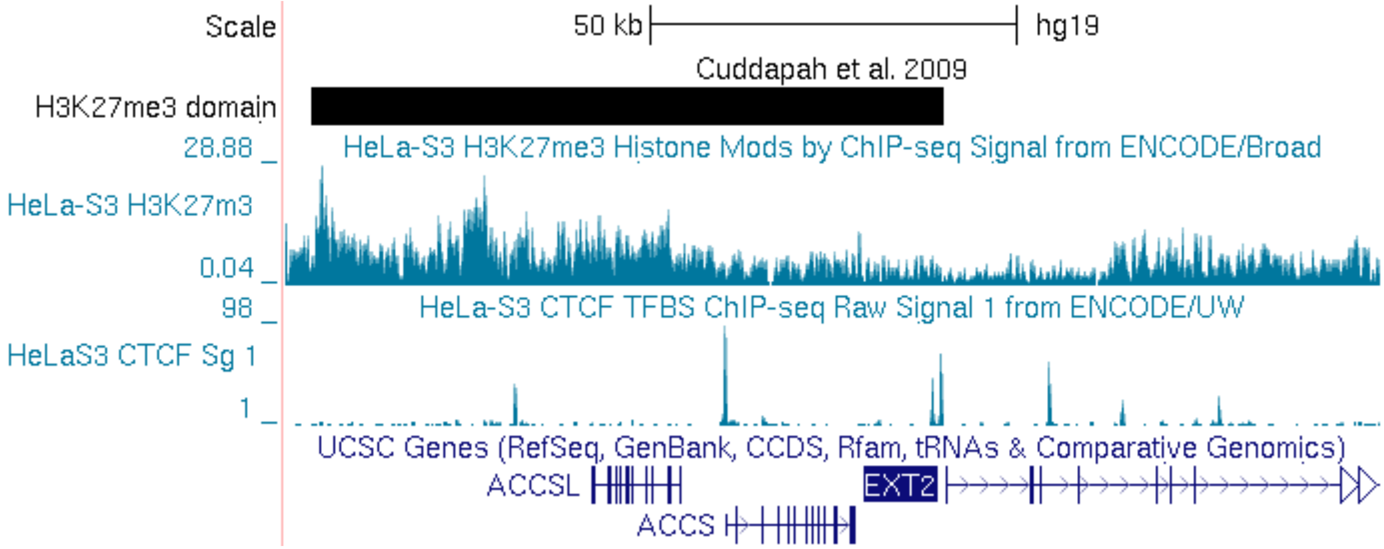

OAS1

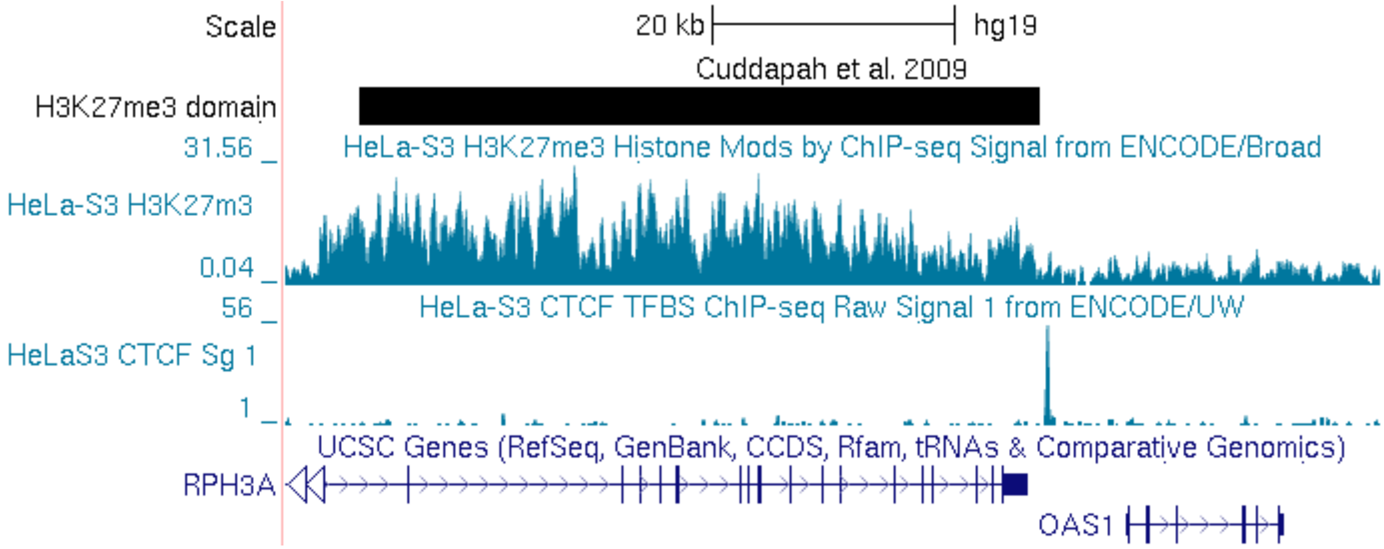

DUSP16

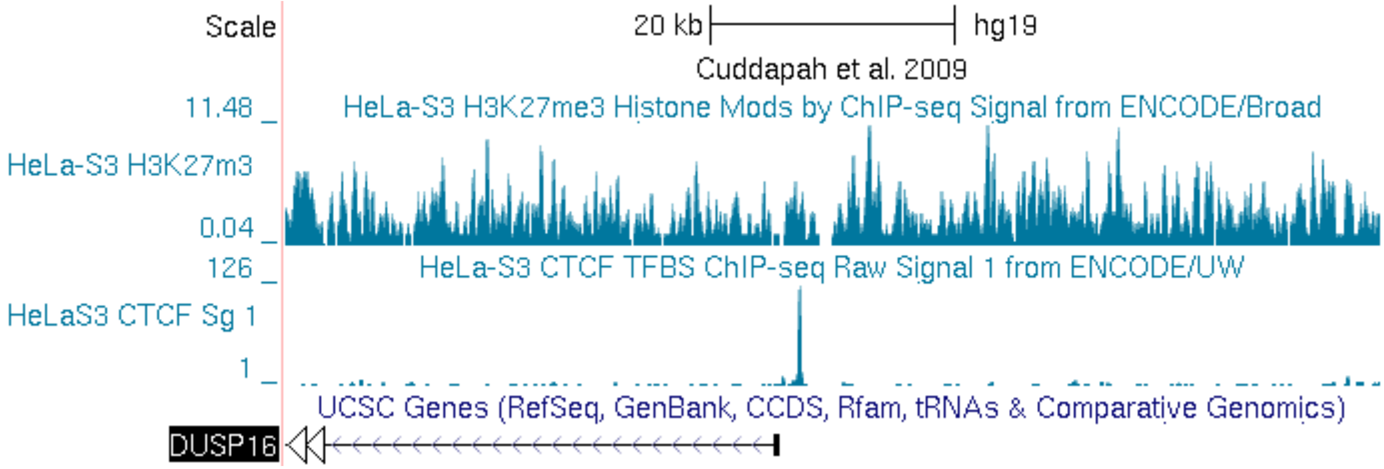

RPLP0

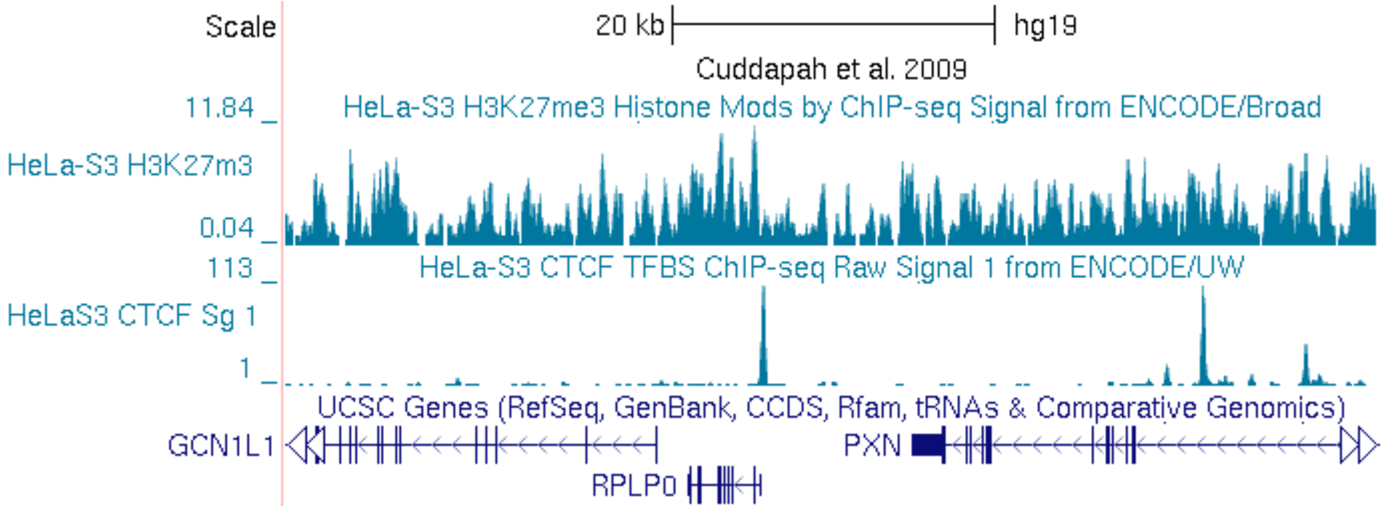

COX6A1

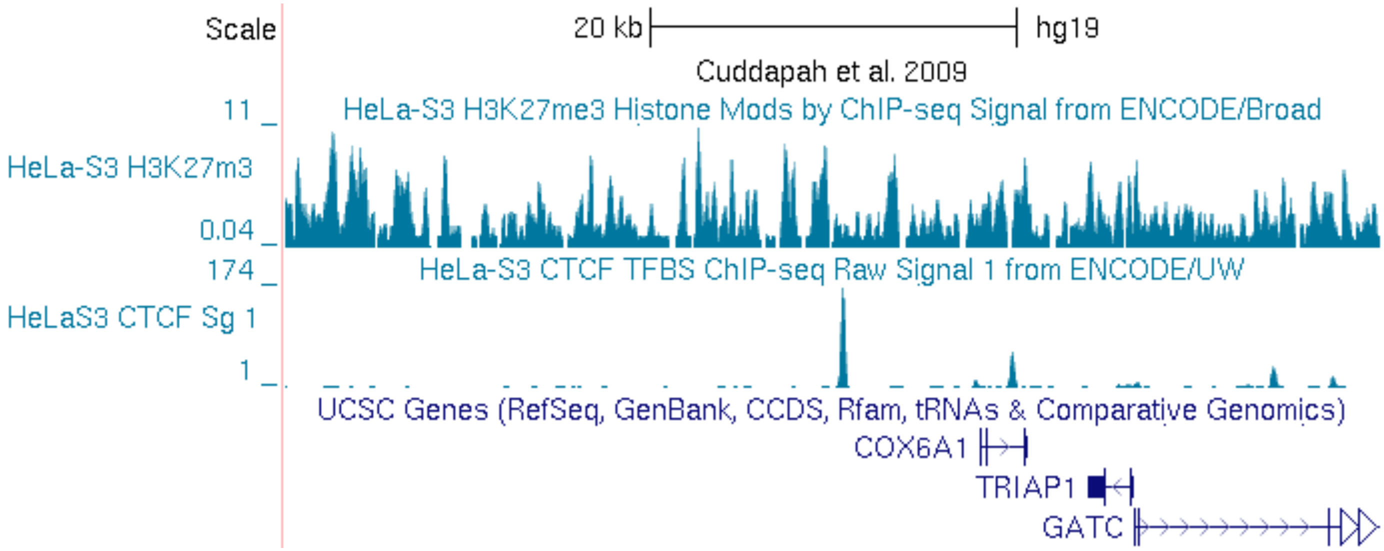

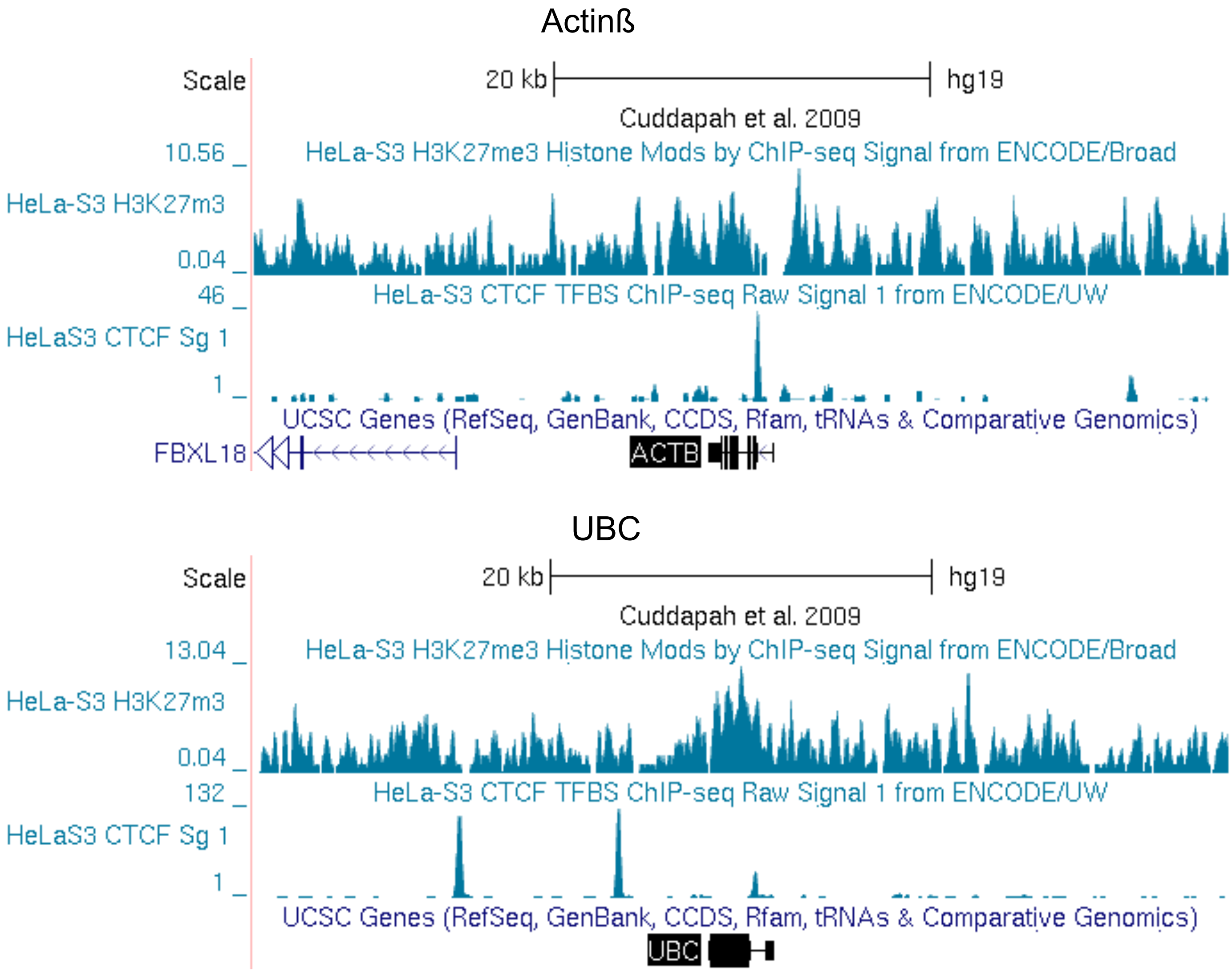

Supplementary Figure S1. UCSC genome browser snapshots. The tracks show from top to bottom the H3K27me3 domain boundaries (Cuddapah et al. 2009), ChIPseq profiles for H3K27me3 and CTCF of HeLa-S3 cells and annotated gene transcripts. Spreading of H3K27me3 was investigated at the CTCF positive domain borders of the genes ATP8B2, EXT2 and OAS1. These are located within active chromatin in close vicinity to a H3K27me3 domain boundary bound by CTCF.

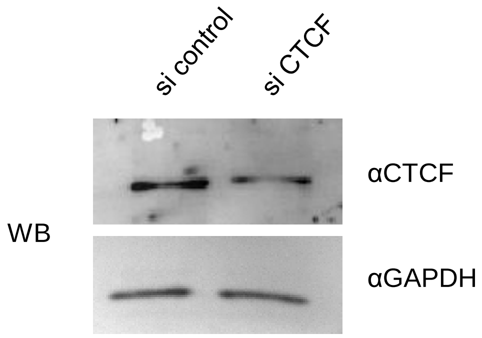

Supplementary Figure S2. CTCF is downregulated in HeLa cells upon siRNA treatment. Hela cells were transfected with si control or siCTCF in 3 biological replicates for detection of protein levels at 6 days post transfection. (Upper panel) Western blot assayed with CTCF (Millipore 07-729) and GAPDH antibody. (Lower panel). Shown is one representative blot with mild reduction of CTCF, which is sufficient to significantly reduce chromatin binding of CTCF as in figure 1.

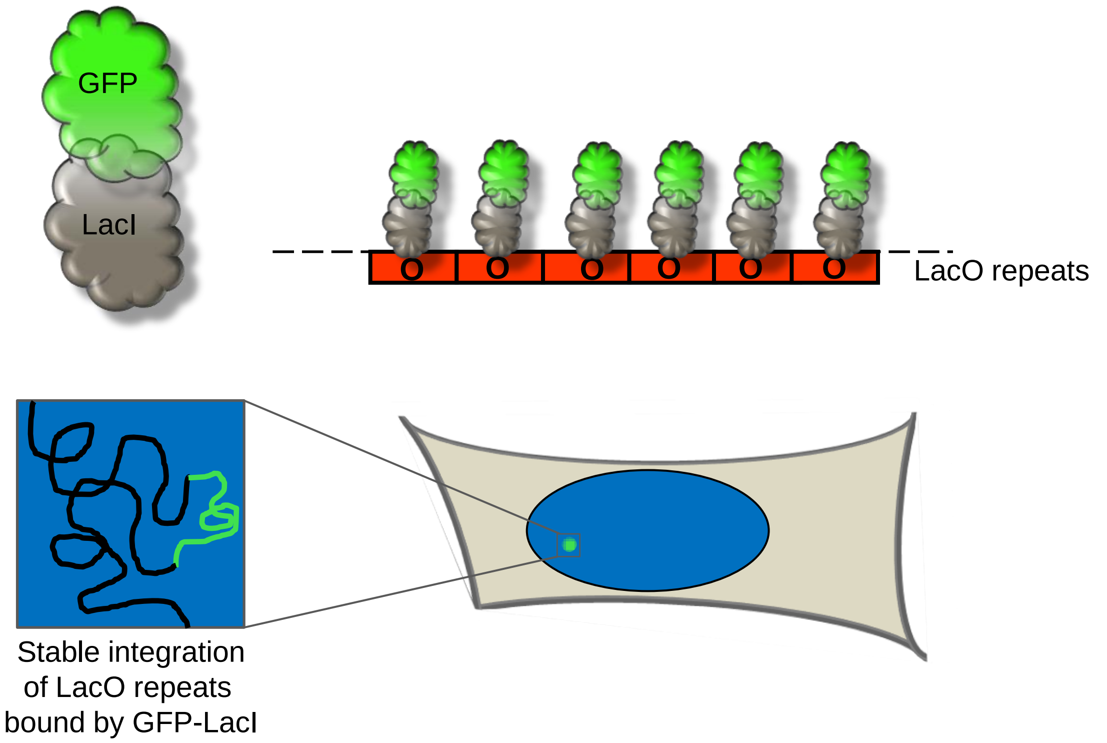

Supplementary Figure S3. Schematic representation of the LacO/LacI system for labeling chromatin in living cells. Arrays of the lacO repeats are integrated into the genome of U2OS cells. The lac repressor LacI binds with high affinity to these arrays. Thereby, transfection of LacI fusion proteins together with GFP visualizes these genomic loci.

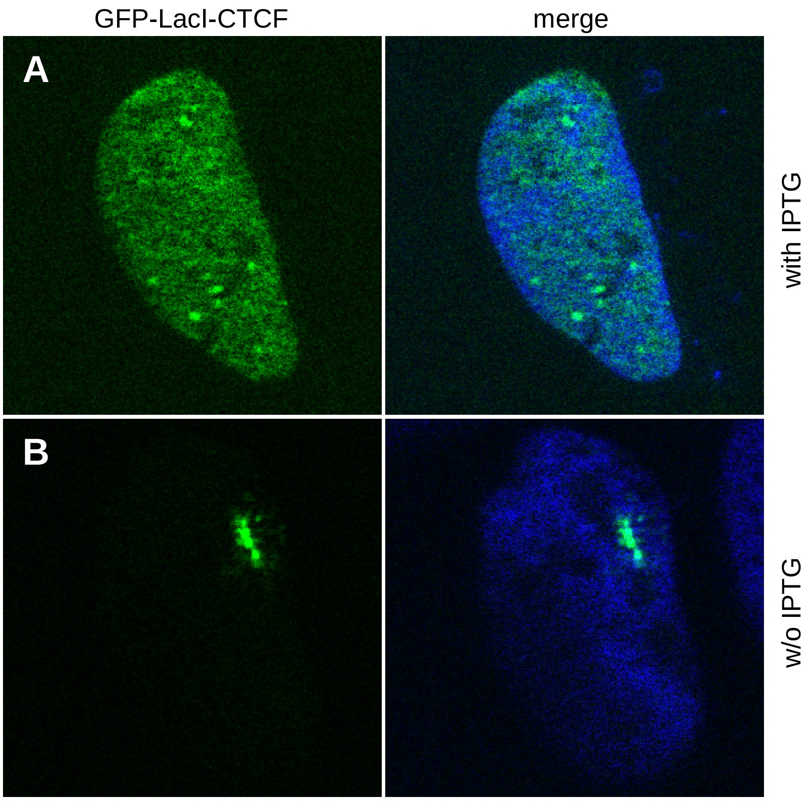

Supplementary Figure S4. IPTG prevents binding of GFP-LacI-CTCF to the array. F42B8 cells were transfected with GFP-LacI-CTCF and incubated for 48h in the presence (A) or absence (B) of 150µM IPTG. The cells were fixed and the nucleus was stained with Hoechst. The left panels show the GFP tagged LacI-CTCF, the right show the merge.

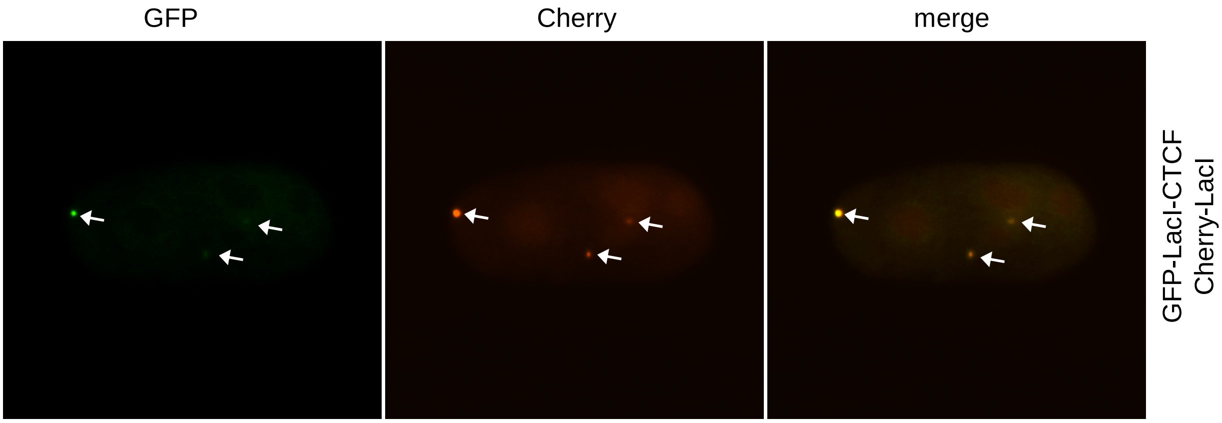

Supplementary Figure S5. Cherry-LacI targets the same area as GFP-LacI-CTCF. F6B2 cells were co-transfected with GFP-LacI-CTCF and Cherry-LacI at a ratio of 1:2. A representative micrograph is shown. Arrows point at the arrays.

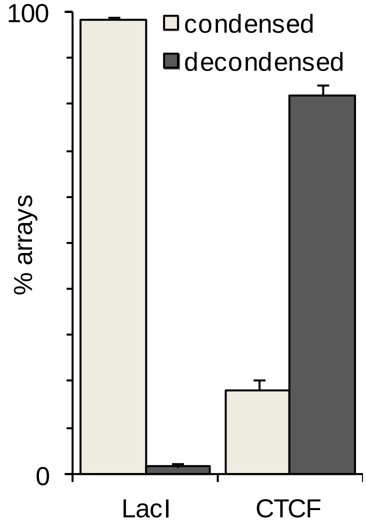

Supplementary Figure S6. More than 80% of the arrays are decondensed upon recruitment of CTCF. F42B8 cells were transfected with either GFP-LacI (LacI) or GFP-LacI-CTCF (CTCF). The arrays were analysed by their size and categorised accordingly.

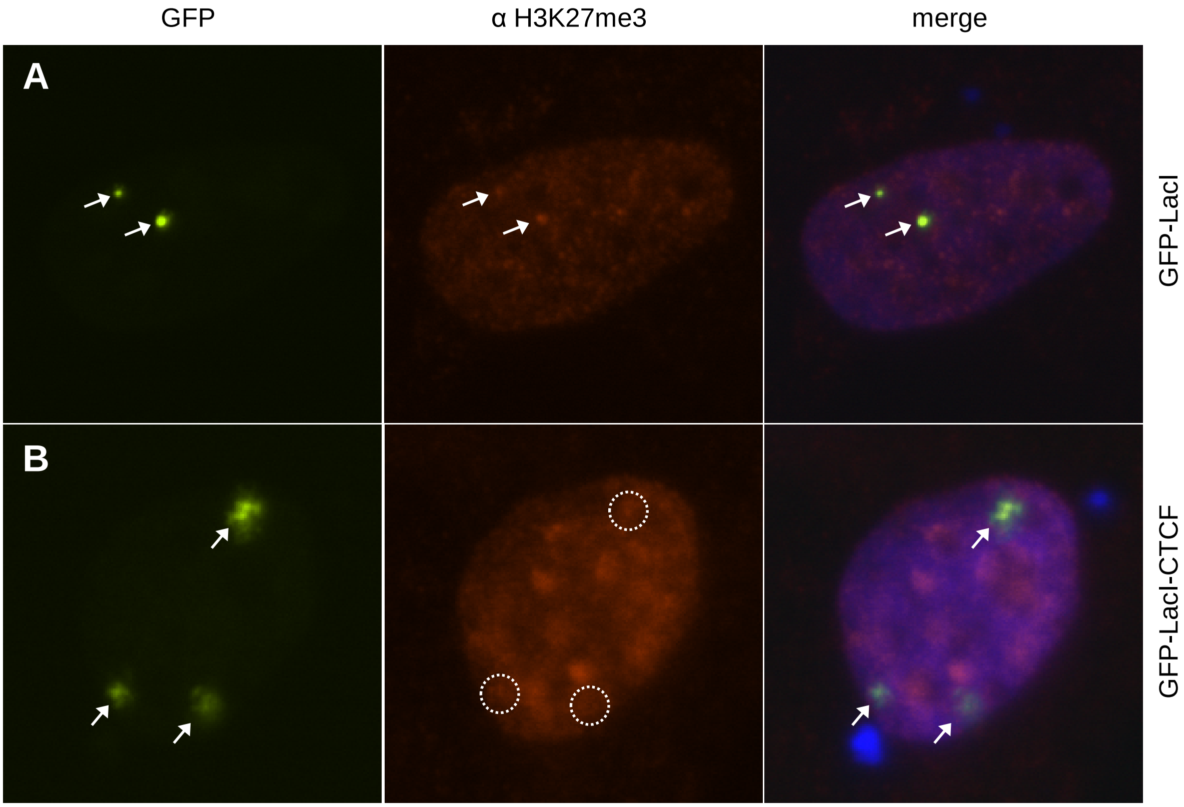

Supplementary Figure S7. Recruitment of GFP-LacI-CTCF in F6B2 cells to heterochromatic LacO sites causes loss of H3K27me3. F6B2 cells were transfected with either GFP-LacI (A) or GFP-LacI-CTCF (B) and incubated for 48h. Fixed cells were treated with an antibody against H3K27me3. The left panels show the GFP tagged LacI or LacI-CTCF, the middle show staining of indicated histone modification by indirect immunofluorescence, the third show the merge. Arrows point at positive signals, whereas circles represent a missing signal.

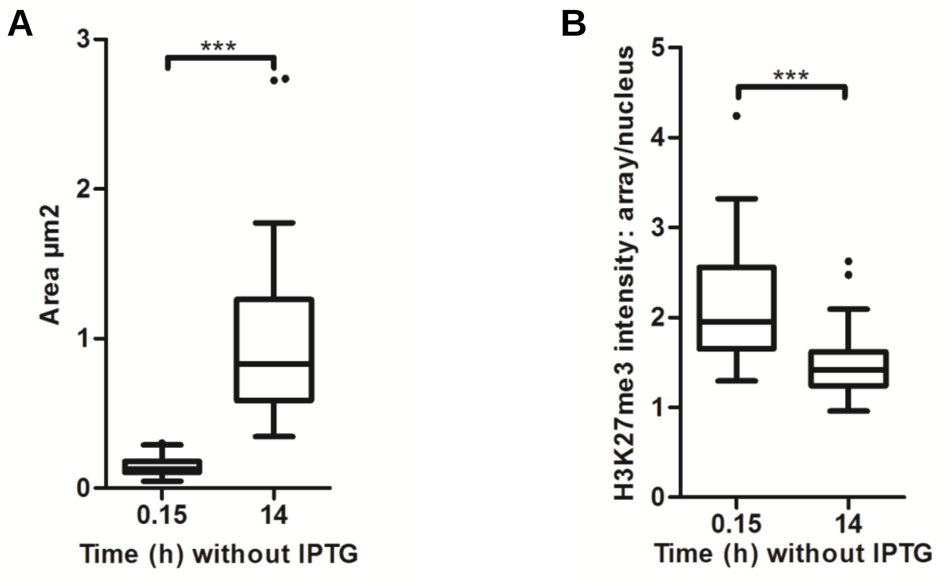

Supplementary Figure S8. Expansion of the array is not cell cycle dependent. F42B8 cells were transfected with GFP-LacI-CTCF in the presence of IPTG and incubated for 24h followed by Colchicin treatment for 5h. Mitotic cells were collected and further cell cycle progression was blocked with Aphidicolin at early S-phase. IPTG was removed from the medium at indicated time points. Immunostaining was performed with an antibody against H3K27me3 and the nucleus was stained with Hoechst. The size in μm^2 (A) and the intensity (B) of antibody staining of arrays was measured by the Volocity software. The size of ~50 arrays was measured and presented as a box plot, with whiskers as defined according to Tukey. Significance was controlled with a two tailed Mann-Whitney-test. P (***) < 0.001

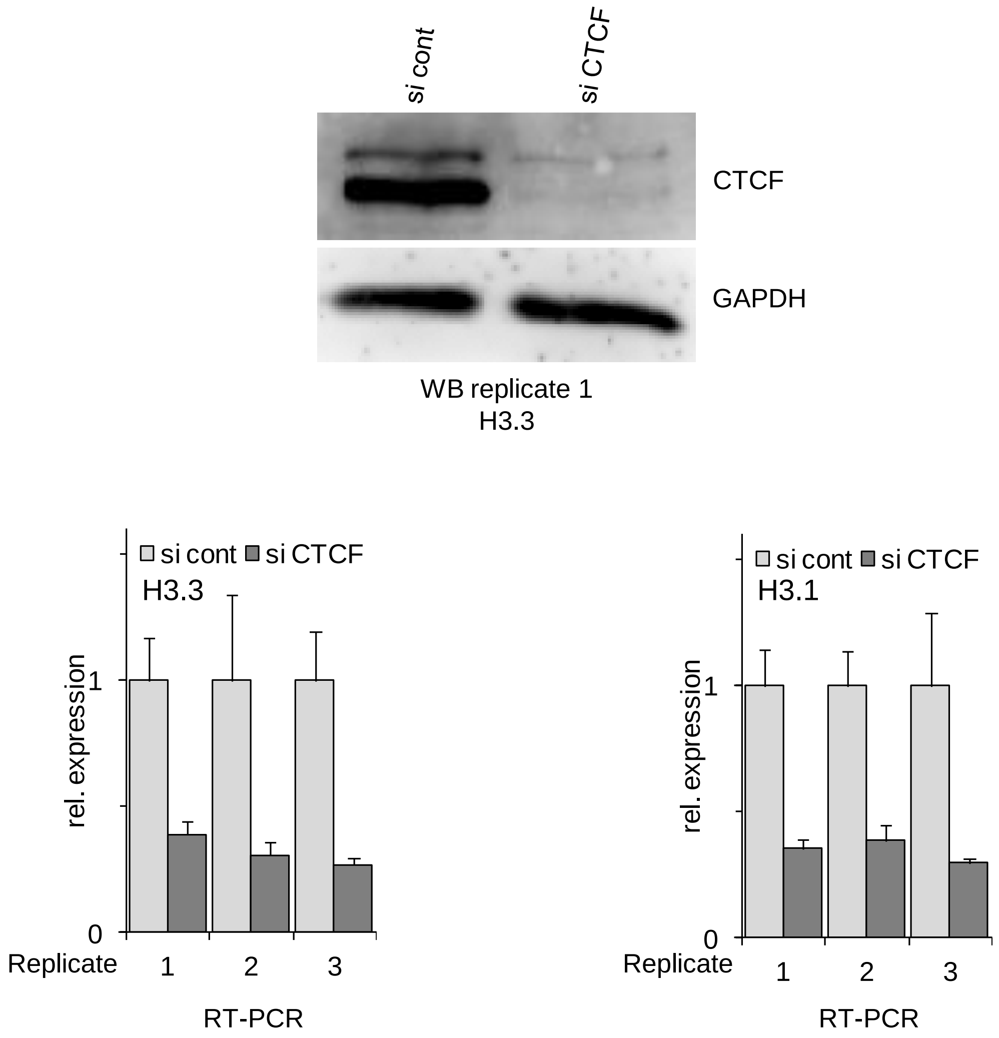

Supplementary Figure S9. CTCF is downregulated in HeLa S3 H3.3-Flag-HA and HeLa S3 H3.1-Flag-HA cells upon siRNA treatment. Hela S3 H3.3 / H3,1 cells were transfected with si control or siCTCF in 3 biological replicates and splitted for detection of protein and RNA levels at 4 days post transfection. (Upper panel) Western blot assayed with CTCF N2.2 and GAPDH antibody. (Lower panel) qRT–PCR of CTCF mRNA relative to GAPDH and UBC.

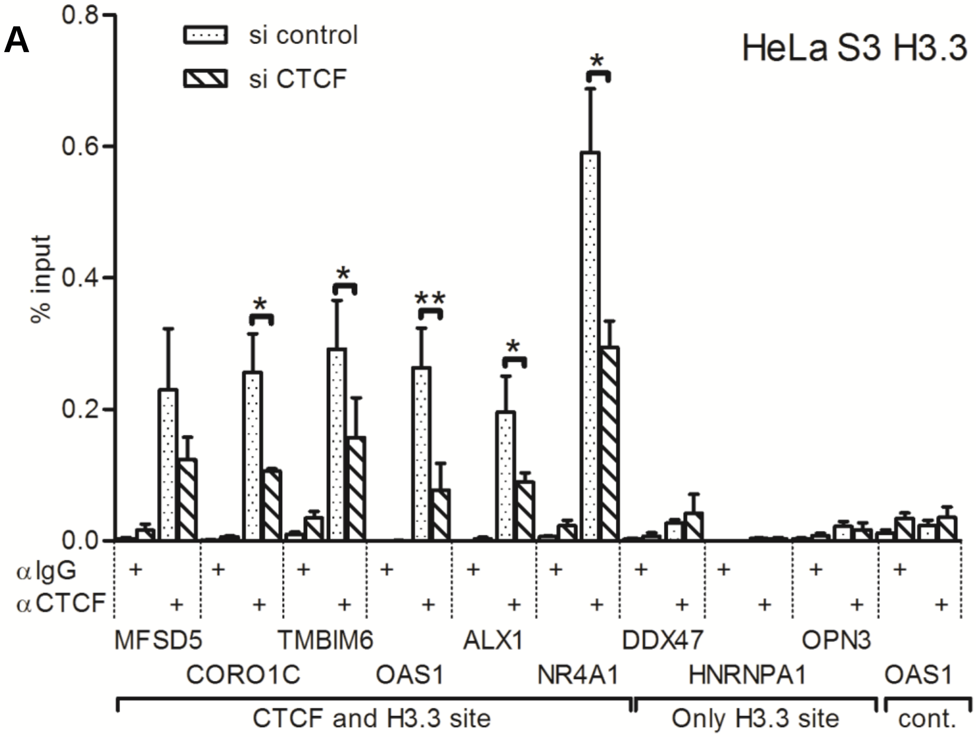

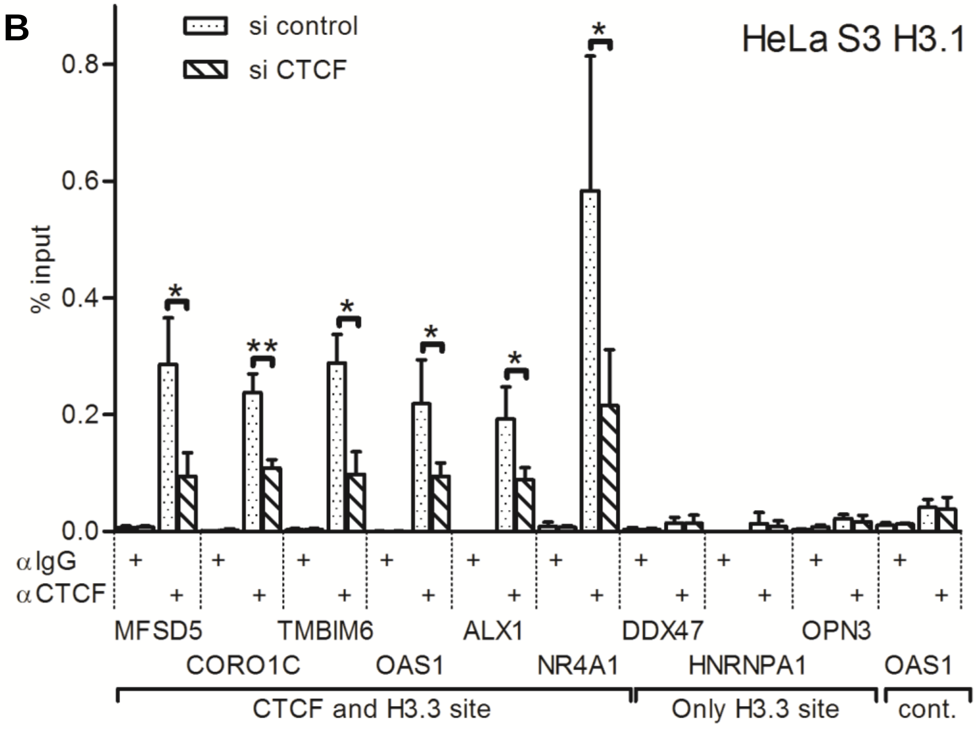

Supplementary Figure S10. CTCF binding to putative target sites in HeLa S3 cells is reduced upon CTCF kd. HeLa S3 cells were treated with siRNA against CTCF or control siRNA. ChIP was performed with CTCF antibody to confirm binding of CTCF in HeLa S3 H3.3 (A) and H3.1 (B) cell clones. Precipitated DNA was PCR amplified with indicated primers and normalised for input material. P-values were calculated by paired two-tailed Student's t-test and asterisks represent values from 0.05 to 0.01(*) and 0.01 to 0.001 (**) respectively.
