# Supplementary material for: CTCF induces histone variant incorporation, erases the H3K27me3 histone mark and opens chromatin
Source: Nucleic Acids Res. 2014 Oct 7;42(19):11941–51. doi: 10.1093/nar/gku937 (PMC4231773; doi:10.1093/nar/gku937)
Supplement: SUPPLEMENTARY DATA [file supp_gku937_nar-03423-x-2013-File011.docx]

**Supplementary Material and Methods**

**ChIP and Native ChIP**

ChIP was essentially done as described (55). Cells were fixed using 37% formaldehyde (CalBiochem) with a final concentration of 1% for 10min. at room temperature. Incubation of 1/7 volume of 1M glycine for 5min. stopped the fixation process. Cells were washed with PBS and harvested in PBS + 1mM PMSF. After centrifugation for 2min. at 2000 rpm at 4°C the supernatant was removed and cells were lysed using 1ml SDS-lysis-buffer (0.5% SDS, 10mM EDTA, 50mM Tris/HCl pH 8.1) supplemented with protease inhibitors (Complete Mini, Roche) per 10^7^ cells for 10min. on ice. Chromatin was sheared to an average size ranging from 400–800bp. After sonification the samples were centrifuged for 10min., 4°C at maximum speed. The supernatant (chromatin) was diluted 1:10 with dilution-buffer (0.01% SDS, 1.1% Triton X100, 1.2mM EDTA, 16.7mM Tris/HCl pH8.1, 167mM NaCl) and 1ml of the dilution was used for each chromatin immunoprecipitation. 10% of the chromatin used for one ChIP was preserved as an input sample and stored at -20°C. The lysate was pre-cleared by rotation at 4°C for 2h using 1ml of the dilution and 20µl ProteinG Plus/ProteinA Agarose (Calbiochem). After centrifugation at 4°C for 1min at 2000 rpm the supernatant was incubated with the corresponding antibody rotating over night at 4°C (H3 ab1791, H3K27me3 Millipore 07-449, CTCF Millipore 07-729, CTCF N2.2 and H3K9ac Upstate 07-352). Binding of the immune-complexes occur afterwards by incubation of the chromatin with 20µl of ProteinG Plus/ProteinA Agarose for 2h at 4°C. After incubation the beads were washed for 5min. rotating at 4°C one time with low-salt-buffer (0.05% SDS, 1%Triton x100, 2mM EDTA, 20mM Tris/HCl pH 8.1, 150mM NaCl), one time with high-salt buffer (0.05% SDS, 1%Triton x100, 2mM EDTA, 20mM Tris/HCl pH 8.1, 500mM NaCl), one time with LiCl-buffer (0.25M LiCl, 1% NP40, 1% Deoxycholat, 1mM EDTA, 10mM Tris/HCl pH 8.1) and two times with TE-buffer (10mM Tris, 1mM EDTA, pH 8.0). Chromatin bound to the beads and input material were resuspended in 100µl TE, 1µl of 10mg/ml RNAse was added followed by an incubation of 30min. at 37°C to digest the remaining RNA. Next 5µl of 10% SDS, 1µl of 20mg/ml Proteinase K was added and incubated for further 2-4h at 37°C. The reverse crosslink was performed by incubation of the samples over night at 65°C. DNA was recovered by using Illustra GFX PCR DNA and gel Band Purification Kit (GE HealthCare) and PCR amplified.

Native ChIP (NChIP)**.** All preparations were done at 4°C if not mentioned otherwise. 10^7^ cells were harvested and washed with ice-cold PBS followed by incubation for 10 min. in 1.6ml lysis buffer (LB: 10mM Tris-HCl pH7.4, 2mM MgCl_2_, 10mM NaCl, 0.5% Triton X-100) supplemented with 10mM PMSF and protease inhibitors (Complete Mini, Roche). Afterwards cells were layered onto 2ml v/v 50% sucrose/LB bed and centrifuged for 10min to pellet nuclei. MNase digestion was performed in 1ml MNase buffer (50mM Tris-HCl pH 8.5mM CaCl_2_, 10mM NaCl, 0.4% TritonX-100) at 37°C such that the major fraction consists of mononucleosomes. The reaction was stopped by adding EDTA to a final concentration of 10mM. After passing the nuclei 15 times through a homogenizer the solution was centrifuged at full speed for 10min and the supernatant was diluted tenfold with IP buffer (10mM Tris pH7.5, 10% glycerol, 300mM NaCl, 0.1% NP40) and 1ml was used per IP (Flag M2 Sigma). Antibody protein A/G bead complexes were washed three times with IP-buffer and twice with TE followed by Proteinase K digestion for 1h at 55°C. DNA amounts were analysed by realtime PCR.

**RNA isolation.** Small interfering RNAs were purchased from Invitrogen (CTCFHSS 173820, CTCFHSS 116456, CTCFHSS 116455, si CTCF 5’-UCACCCUCCUGAGGAAUCACCUUAA-3’(56), si CTCF 5’-GAUGCGCUCUAAGAAAGAA-3’, si control 5’- CUACGAUGAAGCACUAUUATT-3’). Cells were transfected with a mix of specific siRNAs against CTCF and control siRNA according to the manufacturer manual using Lipofectamin^TM^ RNAiMax from Invitrogen on two consecutive days and incubated for a total of 96h. RNA was isolated with the NucleoSpin^®^ RNA II kit (Macherey-Nagel) and transcribed into cDNA (Transcriptor Kit, Roche). Realtime PCR values were normalised to housekeeping genes GAPDH, actin and UBC (57).

**Luciferase assay**. Experiments were performed essentially as described in (58) with minor modifications. In detail, 293T cells were co-transfected with pGL3-LacO, βGal-vector and GFP-LacI constructs using JetPEI^®^ (Polyplus transfection_TM_). After 48h luciferase activity was measured and corrected for βGal expression.

**Western blot.** Western blot was performed as described previously (55). Primary antibodies used were: CTCF N2.2 antibody (rabbit, 1:2000 in PBST containing 5% milk), and GAPDH antibody (1:2000 in PBST containing 5% milk). αGAPDH was purchased from Santa Cruz (FL-335) and αCTCF (N2.2) was generated as described (59) using GST-linked chicken CTCF (amino acids 2–267) fusion proteins.

**FAIRE assay.** F42B8 cells were cotransfected with either GFP-LacI or GFP-LacI-CTCF and pPuro on a 140mm dish followed by incubation in selective media (1µg/ml) for 72h. Formaldehyde was added directly to the plates at room temperature to a final concentration of 1% and incubated for 45 seconds. Glycin was added to a final concentration of 125 mM for 5 minutes to quench the formaldehyde and cells were harvested by scraping into PBS/PMSF. After centrifugation cells were resuspended in FAIRE lysis buffer (2% Triton X-100, 1%SDS, 100mM NaCl, 10mM Tris-Cl, pH 8.0, 1mM EDTA) and sonicated using the Bioruptor (Diagenode) 5 cycles 30 seconds ON/OFF high power at 4°C. DNA was isolated by adding an equal volume of phenol-chloroform, vortexing, and spinning at 14000rpm at 4°C for 5 min. The aqueous phase was isolated and stored in a separate tube. 400µl TE was added to the remaining organic phase and vortexed. The centrifugation was repeated as above and the aqueous phase was combined with the first fraction. A final phenol/chloroform extraction was performed with the pooled fractions to ensure that all protein was removed. The DNA was precipitated with 0.3 M Na-acetate and 20µg/ml glycogen and 2 volumes ethanol at -20°C overnight. After centrifugation, the pellet was washed with 70% ethanol and dried. The pellet was resuspended in water and treated with RNase A (100µg/ml) for 2h at 37°C. The reverse of crosslinking was performed at 65°C overnight. Precipitated DNA was then analysed in qPCR.

**Plasmids.** To generate LacI-GFP fusion proteins the vector pSV2 GFP-LacI (23) was cut with *SmaI.* CTCF full length was cut with EcoR1 from pEGFP-C2-chCTCF (60) blunt ended with T4 polymerase and ligated into the vector backbone. All deletions were PCR amplified, subcloned into pBSK and ligated in the same way. The LacI-Cherry plasmids were similarly generated, but GFP was replaced by mCherry. Human CTCFL (and truncated versions) was PCR amplified from cDNA and ligated into pSV2-mCherry-LacI. CTCF deletion constructs were PCR amplified with corresponding primers and cloned into pSV2-GFP-LacI. The cDNA of H3.3 was isolated from total human cDNA using primers H3.3 s and H3.3 as. The resulting PCR product was digested with EcoRI and SalI and ligated into the vector pEGFP-N1 (Clontech), digested with the same enzymes.

**Primer 5‘-3‘.**

H3.3/H3.1 ChIP

MFSD5 GCTGCCACTGAACTTTAGCC AGTCCTGCCCCTGTCACTTA

CORO1C CAGAGCCCCTCAATACAGGA AGCAGGAGGCTGGTTTAGG

TMBIM6 AGAGGCGGGTTAGGAAGGTA TGCCACTCTTGCTCAGACAG

OAS1 CACAGGAACAGCTGGAGACA CTGGGTTGAGAGGGACTCAG

ALX1 AGCTTTGCTTCCAGTTTCCA AACCCACCCCTTCACTAACC

NR4A1 GCTTTCCGAGGTTCTTTTCC TGCCACTCTTGCTCAGACAG

DDX47 AGCAAAAGTCAGGCTCAAGG CCCTAGCCTAGCAACAGCAG

HNRNPA1 TGGCAAGGGAAAATCAAGTT TTCTGCAACAGATGGTGAGC

ONP3 TGTGGTTGCACAATCCCTAA TAGCTTGCACAAACCCTGTG

OAS1 cont GATTAGGCTAGTGCCCAGGTC GGGGGAGGTAGGCATAGAGA

H3K27me3 ChIP

OAS1 (-8.1) ACAACCACCCAGCATCTTTC AGGACTTCGCAGAAACCAGA

OAS1 (-6.5) TTGATGCCTTGTGATTTCCA TGTCTCCAGCTGTTCCTGTG

OAS1 (-0.1) AATTCAGCACTGGGATCAGG TTCTGACTGAGCTTGGA

OAS1 (+2.0) GGGTGGGAGGAGATCTTAGG TCCCCACATTATGCCAATTT

ATP8B2 (-3.0) TCTGTCTGGTGATGGTGGAA GGTGGGGGATACAAGAGGAT

ATP8B2 (-0.8)ACCAGGACTCAGGCTTCTCA GGTTGTGCCCTACACTGGAT

ATP8B2 (+0.4)GCGTACAGGATGGGAGGAT CCTCAGGCATAGACCTCCAC

ATP8B2 (+2.3)GTCCCTGGGATGGATACCTT GCCTCCTTCTCCCAGTCTCT

EXT2 (-9.5) TTCCACTTCCAGGGTTCAAG TGGTGGCTCATGCCTATGTA

EXT2 (-0.1) GTGGTGTCTCGTTTGGGTTT AGCACCGCTGCAGTGTTTTC

EXT2 (+1.5) ATTCACTTTCCCCGACTTCC TGCCAGGGTCTGTTTTAAGC

F42B8 FAIRE

LacO-Array CGGCTCTAGCAAGACTGCAT ATCCGCATTCCTCCCATACT

OAS1 cont GATTAGGCTAGTGCCCAGGTC GGGGGAGGTAGGCATAGAGA

GAPDH CCATCTCAGTCGTTCCCAAAGTCC GATGGGAGGTGATCGGTGCT

RT

CTCF GCTTGTATGTGTCCCTGCTGGC TTACACGTGTCCACGGCGTTC

ATP8B2 GCTGAGCGCTGAGGGTCT CCTGGAACTGCTCAAAGAGG

EXT2 GAAGTACGCTTCCCAGAACCA GCAGTATTGAGCGATGTGTTACA

Actin CCTCGTAGATGGGCACAGT CCGTCTTCCCCTCCATCGTG

GAPDH CACCACCAACTGCTTAGC GGCATGGACTGTGGTCATGAG

UBC GCAAGACCATCACCCTGGGA CTAACAGCCACCCCTGAGAAC

hCTCFL_cDNA

forward: TTATGGCAGCCACTGAGATCTCT

reverse: CTCTCACTTCATCGTGTTGAGGA

Deletion constructs CTCF:

NCTCFNterm: CAGGCTAGCGAATTCATGGAAGGTGAAGCAGTTG

NCTCFZnF: AAAGAATTCGGTGTAAAGAAGACATTC

NCTCFCterm: GGTGAATTCGGAGAGAATGGAGGAGAG

CCTCFNterm: CTTCTCGAGTCATTTCTTTTTAATTTTTGTTGG

CCTCFZnF: TCCCTCGAGTCATTCCCCACCATCTAGGC

CCTCFCterm: CTTCTCGAGTCACCGGTCCATCATGCTGAG

Deletion constructs CTCFL:

NBORISNterm: CGCGCTAGCGAATTCATGGCAGCCACTGAGATC

NBORISZnF: GGAGAATTCGGAACCTTCCACTGTGATG

NBORISCterm: GAAGAATTCTCGGCTGCTTCAGGAAAGGG

CBORISNterm: GTGGTCGACTCATTTTGCTCCCTTTGTCTTTC

CBORISZnF: TGAGTCGACTCACTTTGCTTCCCCTGATCC

CBORISCterm: GAAGTCGACTCACTTATCCATCGTGTTGAGG

H3.3 s: GGGGGAATTCAGGAGGTCTCTGTACCAT

H3.3 as: GTGGGTCGACAAGCACGTTCTCCACGT

**Supplementary References**

55. Zhang, R., Burke, L.J., Rasko, J.E., Lobanenkov, V. and Renkawitz, R. (2004) Dynamic association of the mammalian insulator protein CTCF with centrosomes and the midbody. *Exp Cell Res*, **294**, 86-93.

56. Wendt, K.S., Yoshida, K., Itoh, T., Bando, M., Koch, B., Schirghuber, E., Tsutsumi, S., Nagae, G., Ishihara, K., Mishiro, T. *et al.* (2008) Cohesin mediates transcriptional insulation by CCCTC-binding factor. *Nature*, **451**, 796-801.

57. Vandesompele, J., De Preter, K., Pattyn, F., Poppe, B., Van Roy, N., De Paepe, A. and Speleman, F. (2002) Accurate normalization of real-time quantitative RT-PCR data by geometric averaging of multiple internal control genes. *Genome Biol*, **3**, RESEARCH0034.

58. Günther, K., Rust, M., Leers, J., Boettger, T., Scharfe, M., Jarek, M., Bartkuhn, M. and Renkawitz, R. (2013) Differential roles for MBD2 and MBD3 at methylated CpG islands, active promoters and binding to exon sequences. *Nucleic Acids Research*, **41**, 3010-3021.

59. Hoogenraad, C.C., Akhmanova, A., Grosveld, F., De Zeeuw, C.I. and Galjart, N. (2000) Functional analysis of CLIP-115 and its binding to microtubules. *J Cell Sci*, **113**, 2285-2297.

60. Burke, L.J., Zhang, R., Bartkuhn, M., Tiwari, V.K., Tavoosidana, G., Kurukuti, S., Weth, C., Leers, J., Galjart, N., Ohlsson, R. *et al.* (2005) CTCF binding and higher order chromatin structure of the H19 locus are maintained in mitotic chromatin. *The EMBO journal*, **24**, 3291-3300.
